# AntID_APP: Empowering Citizen Scientists with YOLO Models for Ant Identification in Taiwan

**DOI:** 10.3390/biology15060470

**Published:** 2026-03-14

**Authors:** Nan-Yuan Hsiung, Jen-Shin Hong, Shiu-Wu Chau, Chung-Der Hsiao

**Affiliations:** 1 Department of Computer Science and Information Engineering, National Chi Nan University, Nantou 545301, Taiwan; s110321901@ncnu.edu.tw (N.-Y.H.); jshong@ncnu.edu.tw (J.-S.H.); 2 Department of Engineering Science and Ocean Engineering, National Taiwan University, Taipei 106319, Taiwan; chausw@ntu.edu.tw; 3 Department of Bioscience Technology, Chung Yuan Christian University, Taoyuan 320314, Taiwan

**Keywords:** biodiversity, taxonomy identification, iNaturalist, ant identification, deep learning, YOLO

## Abstract

Ants are vital bioindicators that contribute to soil health and food webs, making accurate identification essential for biodiversity monitoring and conservation. However, traditional taxonomic methods are time-consuming and require specialized expertise, limiting large-scale data collection and public participation. This paper presents AntID_APP, a web-based application designed to support citizen scientists in Taiwan by enabling real-time, image-based detection and the identification of native ant genera. Fine-tuned YOLO models first detect ants in user-uploaded images and then classify them at the genus level. The models were trained on a curated dataset of 60,429 open-access images from iNaturalist, covering 54 native ant species. To ensure robustness in real-world conditions, we applied targeted data augmentation and evaluated multiple YOLO versions (v9–v12). The best-performing model achieved a mean Average Precision (mAP50: 0.935–0.948, mAP50-95: 0.777–0.807) for the detection task, followed by accurate genus-level identification. The application features an intuitive interface and a lightweight asynchronous server architecture, allowing users to upload images and receive both visual detection results (bounding boxes) and genus predictions efficiently. By combining high accuracy with accessibility, AntID_APP offers a scalable solution for biodiversity monitoring and public engagement in ecological research.

## Simple Summary

Identifying ants is crucial for tracking environmental health, but traditional identification methods require specialized experts and significant time. This makes it difficult for the public to gather ecological data. To address this, we developed a web application to help citizen scientists in Taiwan to instantly identify native ant groups using their photographs. Here, we trained advanced artificial intelligence computer programs on over sixty thousand public images, and our results show that the system can highly accurately locate and identify ants in everyday pictures. However, we also discovered that blurry photos, busy backgrounds, and natural physical differences among ants of the same group can sometimes confuse the program. Regardless, building this tool demonstrated that artificial intelligence is a powerful aid for identifying species, but it still requires careful adjustments for different types of animals and close teamwork between computer scientists and biologists. This accessible tool empowers anyone to participate in scientific research, making large-scale environmental monitoring faster and easier. Ultimately, combining modern technology with public participation will improve how society tracks biodiversity and protects natural ecosystems.

## 1. Introduction

Ant identification is critically important for ecologists due to the multifaceted ecological roles ants play and their sensitivity to environmental changes. First, ants are exceptional bioindicators of ecosystem health. Different species respond uniquely to variations in climate, habitat structure, pollution, and disturbance. By monitoring ant communities—including species composition, relative abundance, and distribution—ecologists can rapidly assess an area’s ecological integrity. Shifts in ant populations often signal broader environmental changes, serving as an early warning for ecosystem degradation or recovery. Second, ants significantly contribute to soil health through foraging and nest-building activities that aerate the soil, enhance water infiltration, and redistribute nutrients. Since different ant species affect soil properties differently, accurate identification helps ecologists decipher the relationship between ant presence and soil fertility. Third, ants are integral components of food webs, functioning as predators, scavengers, and herbivores. Accurate identification is essential for mapping trophic interactions and understanding energy flow within ecosystems.

In biodiverse regions like Taiwan, which hosts rich and potentially endemic ant fauna, accurate identification enables proper species cataloging, assessment of conservation status, and targeted protection for vulnerable populations. Furthermore, rapid and accurate identification of invasive species—such as the red imported fire ant—is the critical first step in implementing containment and eradication programs. However, traditional species identification relies on specialized taxonomic expertise and is often time-consuming, labor-intensive, and prone to errors, creating a bottleneck for large-scale biodiversity monitoring.

**Figure 1:**
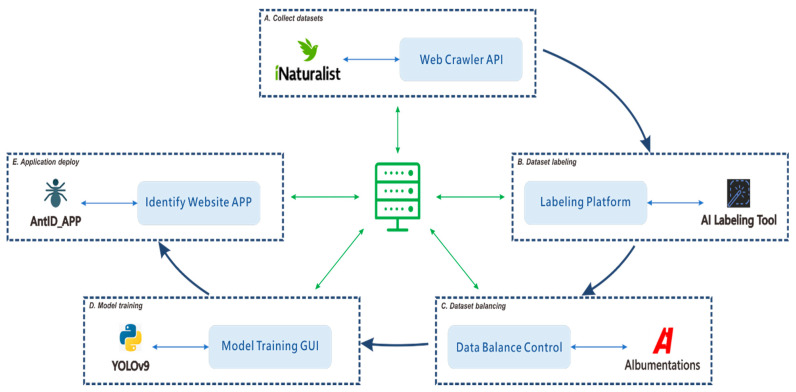
Modular workflow for developing an automated species identification system using a YOLO-based deep learning model. The workflow integrates five core modules: (**A**) collecting confirmed species images via the iNaturalist Web API; (**B**) developing a collaborative platform for YOLO-compliant dataset annotation; (**C**) balancing the dataset through augmentation or reduction to mitigate class imbalance; (**D**) implementing a control interface for streamlined hyperparameter tuning and model testing; and (**E**) automating the deployment of optimized models to the web application. This modular architecture is the foundational step toward an automated production pipeline. The diagram depicts high-level concepts with interdependent, non-linear processes, where execution paths can form various sub-threads (e.g., (**A**) → (**B**) → (**D**) or (**B**) → (**C**) → (**D**)). Effectively managing this coordination demands substantial research investment and resource allocation.

Machine learning has emerged as a promising solution to these challenges, enabling the development of AI-powered automated identification systems that can process images rapidly and consistently. Such systems also facilitate broader participation by citizen scientists, allowing non-experts to contribute to biodiversity research. YOLO first introduced by Redmon et al. in 2016 [1], that has evolved through multiple iterations, each optimized for different scenarios. In our study, we employ an object detection training paradigm that localizes targets by predicting bounding box coordinates, with each box assigned a classification label. The detection results provide the highest-confidence classification for each identified object. For our implementation, we use the YOLOv9 model proposed by Wang et al. in 2024 [2]. Our primary objective is to facilitate interdisciplinary AI research by developing a comprehensive, modular framework for supervised learning-based, genus-level classification capable of supporting automated identification across multiple species. The modular architecture establishes all processes within a loosely coupled system framework; the complete modular workflow of this study is illustrated in Figure 1.

The remainder of this paper is organized as follows. Section 2 presents a state-of-the-art literature review; Section 3 is the Materials and Methods section; Section 4 reports the experimental results; Section 5 discusses these results; Finally, Section 6 concludes the paper. The AntID_APP (https://webs.cloud.ncnu.edu.tw, accessed 3 December 2025) is publicly accessible for readers interested in exploring the system.

## 2. State-of-the-Art Literature Review

The integration of artificial intelligence with citizen science platforms has significantly accelerated biodiversity data collection and species identification. Large-scale initiatives such as iNaturalist and Pl@ntNet [3] have demonstrated the power of combining crowdsourced observations with deep learning models to engage the public in scientific research. Hogeweg et al. [4] developed a large-scale multi-source species identification model trained on 45.4 million images across 41,014 taxa, achieving significant accuracy improvements for rare species through imbalance mitigation techniques. Similarly, platforms like eBird [5], FrogID [6] and Ribbit [7] have successfully employed machine learning for bird and frog call recognition, enabling automated validation and reducing expert workload. These systems highlight the potential of AI to broaden public participation in biodiversity monitoring while maintaining scientific rigor. The LifeCLEF series of challenges has provided standardized benchmarks for species identification since 2011, fostering collaboration among AI experts, citizen scientists, and ecologists [8]. Recent editions address emerging challenges including few-shot classification for rare species, multi-label identification in complex scenes, and individual animal re-identification.

Several reviews have synthesized the growing literature on AI-based insect identification. Gao et al. [9] reviewed 32 studies since 2011, documenting model architectures, taxonomic categories, dataset sizes, and evaluation metrics. Hasan et al. [10] examined 37 studies on crop pest classification (2018–2025), noting a shift from CNNs to hybrid and transformer-based models, while identifying persistent challenges including imbalanced datasets, small pest detection, and edge deployment constraints. Large-scale systems like AInsectID [11] and InsectNet [12] now offer user-friendly interfaces using transfer learning and end-to-end pipelines. Specialized tools such as SpiderID_APP [13] demonstrate the feasibility of region-specific, YOLO-based identification for Taiwanese spiders. For arthropod taxa, Lakyiere et al. [14] reviewed 52 studies on ML-based mosquito identification (2000–2024), highlighting feature extraction for fine-grained traits and revealing limitations—limited dataset diversity, inconsistent preprocessing, and geographic disparities—that parallel challenges in ant identification.

Despite the ecological importance of ants as bioindicators, dedicated AI systems for ant identification remain scarce. Palazzetti et al. [15] introduced AntPi, a Raspberry Pi-based edge-cloud system using YOLO for real-time ant detection. Trained on 1253 manually annotated images across six ant classes, it demonstrated the feasibility of low-cost edge deployment while integrating environmental sensors. Apeinans et al. [16] evaluated YOLOv8 for ant detection and highlighted critical dataset transferability issues: models achieved 98–99% mAP50 on the ANTS dataset but dropped to 5–6% on a domain-specific “WildAnts” dataset. This dramatic performance degradation underscores the need for region-specific, ecologically representative training data—a challenge directly addressed by our Taiwan-focused AntID_APP, which uses locally sourced iNaturalist data. Beyond species identification, Zhang et al. [17] applied an improved YOLOv7 with SENet attention to detect ant nests in drone images of dikes, achieving 91.5% mAP for infrastructure protection, demonstrating the technology’s extension to conservation and engineering domains.

The YOLO family has gained widespread adoption in ecological applications due to its balance of speed and accuracy, with studies benchmarking versions v5 through v12 for tasks from general insect monitoring to specific pest detection. Sorbelli et al. [18] employed YOLO for drone-based monitoring of Halyomorpha halys in orchards, addressing domain shift by creating an ad hoc dataset and analyzing image quality factors. Shaowei et al. [19] combined Latent Diffusion Inpainting (LDI) augmentation with an improved YOLOv12n for rice leaf disease detection, achieving 88.3% mAP50 and demonstrating generative techniques for addressing data scarcity. Santoro et al. [20] compared citizen science classification to two AI classifiers (EfficientNet and DeepFaune) using 51,588 expert-labeled camera trap images, revealing that while AI offers unmatched speed, citizen science can reduce errors. Critically, their simulation identified precision as the strongest predictor of occupancy model accuracy, with lower precision substantially increasing root mean square error.

A comprehensive overview of the related literature is provided in Supplementary Table S1. The literature review reveals several research gaps that motivate the present study: (1) Geographic and taxonomic coverage: Existing ant detection datasets focus on European or generalist species, with no dedicated system for Taiwan’s diverse ant fauna. (2) Rare species handling: Many studies exclude underrepresented taxa due to data scarcity, potentially biasing models against ecologically significant rare genera. (3) Deployment-oriented evaluation: Prior work often emphasizes model performance metrics without sufficient discussion of system architecture, user experience, and scalability for citizen science platforms. (4) Data augmentation trade-offs: While augmentation is commonly used to address class imbalance, the optimal balance between synthetic variation and biological authenticity remains underexplored.

AntID_APP addresses these gaps by: (1) developing the first YOLO-based ant identification system specifically for Taiwan’s 54 native genera; (2) proposing strategies for handling rare taxa through aggregation approaches; (3) implementing a lightweight asynchronous web architecture optimized for real-world citizen science use; and (4) systematically evaluating the impact of data augmentation on per-genus accuracy to ensure ecological validity.

## 3. Materials and Methods

### 3.1. Collect Datasets

Acquiring high-quality datasets for automated identification training poses significant challenges. This process demands large numbers of clearly visible, taxonomically accurate images, yet many biological species—particularly at the genus level—lack sufficient photographic documentation. This scarcity is exacerbated by limited access to taxonomic experts who can verify species classifications, which means most images sourced from the web lack reliable taxonomic metadata. To address these issues, our study utilizes the authoritative open-source platform iNaturalist (https://www.inaturalist.org/, accessed 5 April 2025) as our primary image source. This platform offers distinct advantages: (1) consistent image quality, (2) expert-verified identifications, and (3) continuous taxonomic updates that correct classification errors. These features make iNaturalist an exceptional resource for biological identification training. Notably, we deliberately avoid conventional web scraping methods due to three critical limitations: (1) inefficient time expenditure on fruitless searches, (2) potential classification as malicious activity by website administrators, and (3) risk of IP blocking from excessive traffic. Effective dataset acquisition and cost management remain fundamental considerations for any AI research initiative.

This study employs the iNaturalist API (Application Programming Interface) to facilitate data exchange between our application and their database [21]. The API responds to each request with data in JSON (JavaScript Object Notation) format [22]—a lightweight, human-readable standard widely adopted in web applications due to its ease of parsing and modification. To systematically retrieve all required genus- and species-level image datasets, we developed a five-step protocol: (a) Taxon ID Retrieval: Input family or genus names to obtain corresponding taxonomic IDs. (b) Species Enumeration: Use family/genus IDs to retrieve all species IDs and scientific names. (c) Observation Collection: Query species IDs to obtain all observer IDs. (d) Image Metadata Extraction: Retrieve image IDs, URLs, and annotations using observer IDs. (e) Image Download: Acquire image files through the obtained URLs. Using Formicidae (the ant family) as a case study, Supplementary Table S2 presents exemplary web links and detailed explanations for each step.

### 3.2. Dataset Labeling

The quality of data labeling directly dictates the accuracy of the final model. To enhance efficiency and reduce labor costs, this study developed a dedicated collaborative annotation website. This platform expands the pool of available annotators and incorporates an incentive mechanism to improve both the speed and precision of the labeling process. Annotation is a repetitive and meticulous task that requires precise marking of each object’s location and classification. This challenge is particularly pronounced for eusocial organisms like ants, which exhibit caste divisions, overlapping generations, and other collective behaviors [23]. These traits often result in a high degree of object overlap within images and significant morphological variation among individuals of the same species, further increasing annotation difficulty.

Our collaborative website is built upon the freely available YOLO AI Labeling Tool v1.0.10, developed by LILIN [24]. This software runs locally on users’ machines, which reduces server load and increases processing speed. It includes efficiency-enhancing features like drag-and-drop functionality and batch image processing. The workflow on our website involves splitting the full image dataset into manageable batches (e.g., 1000 images each). Annotators download a batch, label the images locally using the tool, and then upload the resulting TXT annotation files. This batch-based approach facilitates participation from multiple volunteers while preventing individual workload fatigue that could compromise efficiency. Given that species-level classification demands advanced taxonomic expertise, this study focuses on genus-level identification.

Prior to annotation, we conducted a genus-level analysis to balance the dataset. This involved excluding genera with an insufficient number of samples for effective training and downsampling images from genera that exceeded project requirements. We implemented a three-stage annotation workflow: (1) Initial Labeling: A small, balanced subset of images from all target genera is manually annotated. (2) Pre-labeling Model: This initial labeled data is used to train a preliminary ant family (Formicidae) classification model. (3) Model-Assisted Verification: The preliminary model detects and pre-labels the remaining unannotated data, which is then verified manually. For large-scale classification tasks, these three stages can be iterated or executed in parallel to optimize the trade-off between cost-efficiency and annotation accuracy.

### 3.3. Dataset Balancing

Before annotating the dataset, we conducted the systematic filtering of images across all genera. This study established a benchmark of 1000 images per category for YOLO training. For example, the Camponotus genus initially contained 16,951 images—far exceeding the requirement—necessitating a substantial reduction to minimize annotation costs. The retained images were carefully curated to preserve diversity and prevent homogeneity. At the other extreme, the Leptanilla genus contained only two images, which would have required a 500-fold augmentation to meet the training benchmark. However, as demonstrated by Kumar et al., excessive data augmentation can lead to underfitting when dataset homogeneity limits the model’s learning capacity [25]. Overly uniform data from such augmentation compromises model generalizability, causing it to learn augmentation artifacts rather than authentic features. Consequently, Leptanilla was excluded from training due to fundamentally insufficient data. This balancing of image quantity per category is critical for subsequent model accuracy.

According to the official YOLO documentation by Ultralytics, achieving high reliability typically requires at least 100 images per class trained over 100 epochs, with complex tasks potentially needing thousands of images per class [26]. The default training iteration is 300 epochs, which should be reduced if overfitting occurs or increased for underfitting. Our dataset balancing strategy involved setting a maximum data augmentation multiplier between 15 and 20. We analyzed the distribution of the collected image dataset, which covered 71 genera of Taiwanese ants, and excluded 17 genera with fewer than 50 images. Data augmentation was then applied to the remaining 54 genera that had fewer than 1000 images each. Although the subjects (ants) were small, the iNaturalist images were rigorously screened to minimize issues like blurriness, defocus, distortion, or poor exposure.

In ecological monitoring, rare or less common genera are often critical for biodiversity assessments. Although our system has indicated that classifications with confidence scores below 0.6 exhibit higher misclassification rates, to avoid introducing a “forced choice” bias for users, a short-term solution can be implemented at the application level by automatically classifying low-confidence predictions into an “Other/Rare” category. In the long term, to fundamentally address and mitigate the limitation of low resolution within the “Other/Rare” category, a user-centered interface design can complement the model’s predictive capabilities. When an image is classified as “Other/Rare”, the application would not simply return a label. Instead, it would prompt users with the following options: (1) consult a visual guide of rare genera for potential self-identification; (2) submit the image for expert review within the platform; or (3) flag the observation for future dataset expansion. This human-in-the-loop approach ensures that images of rare genera are not simply discarded or misclassified but are appropriately handled, thereby enhancing user engagement and improving the long-term quality of the dataset.

Furthermore, the downloaded images were pre-classified by size, and those where the subject occupied too small a proportion were filtered out during annotation, significantly reducing preprocessing time. Shorten et al. discussed data augmentation techniques for deep convolutional neural networks and how to avoid overfitting [27]. They emphasized carefully evaluating each augmentation method to prevent introducing defects or amplifying biases during training. Traditional data augmentation techniques, including basic operations like rotation, flipping, scaling, and cropping, are widely used in computer vision. For more complex needs, Buslaev et al. proposed Albumentations in 2020—an open-source Python library (Version 3.9.12 was used in this study) that offers a simple and efficient solution [28]. It provides 60 pixel-level and 49 spatial-level transformation methods, which can be randomly combined for image enhancement. The Albumentations team (https://github.com/albumentations-team/albumentations/, accessed 11 February 2025) includes an online real-time demo in their documentation, allowing users to quickly evaluate the effect of each method. Each transformation features adjustable parameters to control intensity or probability, and spatial-level transformations automatically generate adjusted annotation files.

To mitigate the data leakage risks inherent in iNaturalist datasets, we implemented an observer-based split strategy. All images uploaded by a given iNaturalist user were assigned exclusively to either the training, validation, or test set. This prevents the model from learning photographer-specific biases (e.g., background, lighting, camera settings) and ensures that performance metrics reflect generalization to unseen users rather than overfitting to familiar photographic styles. The final split allocated 90% of users to the training set, 5% to the validation set, and 5% to the test set, resulting in 54,000 training images, 2700 validation images, and 3729 test images. We also applied an observation-level deduplication step. For each iNaturalist observation (which may contain multiple images of the same specimen), we randomly selected a single representative image to include in the dataset. This prevents near-duplicate images from appearing across different dataset splits, further reducing the risk of artificial performance inflation. However, for genera with insufficient training data, we retained all available observations to maximize sample size. As a result, some residual leakage risk may persist for these underrepresented taxa.

### 3.4. Model Training

The YOLOv9 model was trained on hardware featuring an NVIDIA A100 GPU (40 GB PCIe) with NVIDIA-SMI 470.86 and CUDA 11.4, using Python 3.9.13 as the core software environment. YOLOv9 provides multiple model sizes (T, S, M, C, E) to balance model complexity, computational cost, and accuracy. Its key innovation is the Generalized Efficient Layer Aggregation Network (GELAN), a lightweight architecture designed to improve information integration and transmission efficiency without sacrificing accuracy. Complementing this, the Programmable Gradient Information (PGI) technology offers adjustable gradient control to enhance model convergence speed and stability. Together, these advancements address long-standing challenges of information loss and computational inefficiency in object detection, setting new benchmarks for performance. For context, the newer YOLOv12 model, introduced by Tian et al., incorporates further enhancements like an Area Attention module (A^2^) and a Residual Efficient Layer Aggregation Network (R-ELAN) to reduce reliance on traditional CNN architectures and alleviate memory bottlenecks [29].

This study initially employed the YOLOv9 GELAN-C model for testing. The training process generates weight files and CSV files containing comprehensive evaluation metrics. These include three loss functions (box_loss, cls_loss, dfl_loss) for both training and validation sets, standard object detection metrics (precision, recall, mAP50, mAP50-95), and learning rate parameters (lr0, lr1, lr2). Upon training completion, the model produces several diagnostic plots: F1_curve, PR_curve, P_curve, and R_curve, which illustrate the relationship between accuracy and detection thresholds. A confusion matrix is also generated, providing statistics on true positives, true negatives, false positives, and false negatives for each category, alongside label distribution plots (labels and labels_correlogram). These metrics and visualizations are critical for model evaluation and hyperparameter tuning. The progressive improvements across YOLO versions, from v3 to v12, in both accuracy and computational efficiency have been comprehensively demonstrated by Jegham et al. [30].

Supplementary Table S3 summarizes several leading models and their key techniques for comparison. When selecting an image recognition model, it is essential to consider scenario-specific requirements, including computing budget (e.g., access to A100/H100 GPUs), latency constraints (e.g., sub-10 ms response time), and data characteristics (e.g., long-tail distribution or class imbalance). However, limited computational resources often lead to prohibitively long training times, and the substantial storage demands of large image datasets further complicate multi-species recognition, necessitating careful resource management. The selection of YOLO as the core detection framework was motivated by several factors relevant to our citizen science application. First, YOLO’s single-stage architecture enables real-time inference, which is critical for maintaining user engagement in interactive applications. In contrast, two-stage detectors such as Faster R-CNN (Girshick et al., 2015) [31] achieve high accuracy but at the cost of slower inference speeds, making them less suitable for latency-sensitive web deployment. Second, YOLO’s unified architecture simplifies deployment across multiple backends (CPU/GPU) and facilitates the ensemble approach used in AntID_APP. Third, recent YOLO versions have demonstrated reliable performance on fine-grained visual classification tasks while maintaining computational efficiency (Wang et al., 2024) [2]. Compared to EfficientDet (Tan et al., 2020) [32], which requires compound scaling adjustments, YOLO’s architectural variants offer more straightforward trade-offs between speed and accuracy for our specific use case.

### 3.5. Application Deploy

Platforms like iNaturalist leverage smartphone cameras and GPS to enable users to quickly record videos, log locations, and document ecological observations—including species, timestamps, and geographical data—anytime and anywhere [33]. This study proposes a distributed service framework, the most cost-effective and practical method for executing object detection across diverse architectures while maintaining seamless web integration is an asynchronous agent model [34]. This architecture mediates between user inputs and YOLO’s computational outputs via server-side web services and database processing. Its key advantage is the asynchronous execution of detection scripts, which prevents performance degradation on web or mobile clients. Furthermore, it supports multi-agent task parallelism or distributed server architectures, allowing research institutions to retain full control over model weights while autonomously managing training, detection, fine-tuning, and upgrades. This ensures both an optimal user experience and framework stability. For third-party integration, conventional polling and webhook methods each have distinct strengths and weaknesses in managing data exchange [35]. Our solution employs a database as an intermediary communication layer, creating a Long Polling-like mechanism where requests persist until data is available, effectively balancing real-time responsiveness with system compatibility.

AntID_APP allows flexible deployment of different models and weights without full-system redeployment. All model paths and parameter configurations are stored in a database and can be modified directly through the collaborative web interface’s admin panel. The system uses API-based protocols for database operations, facilitating communication between collaborators and developers while improving implementation efficiency and enhancing data security. In line with API security recommendations by Díaz et al., we implement authentication, authorization, and encrypted monitoring to ensure information security across institutions [36]. The database features a distributed design [37] with synchronization mechanisms to provide greater flexibility, performance, and resource sharing for collaborative development [38,39]. For managing detection output files in distributed systems, options include local storage at partner institutions with synchronization, or cloud-based solutions like Amazon S3 or Google Drive, both of which require custom API implementations for file access [40].

## 4. Results

### 4.1. Dataset Collection and Analysis

This study developed Python source code to interact with the iNaturalist API. The data collection program accepts two execution parameters: the first specifies one of four functions (species, observations, photos, or download), and the second provides the target genus or family name. The functional workflow is as follows: (1) Species: Retrieves all species IDs for a given genus or family. (2) Observations: Obtains all observation IDs using the species IDs from the previous step. (3) Photos: Acquires all image download URLs using the observation IDs. (4) Download: Downloads all image files from the collected URLs. Although a single, continuous execution would be more efficient, the iNaturalist platform enforces IP-based connection limits that can terminate API sessions. Therefore, the program was designed to run in segmented operations, with the ability to automatically resume from the last recorded ID after an interruption. To address Python version compatibility, we adopted the established solutions of virtual environments [41] or the Anaconda toolkit [42], as supported by Li et al. [43]. Supplementary Figure S1 shows the program’s output, demonstrating how datasets can be exported to files or stored directly in a database.

**Figure 2:**
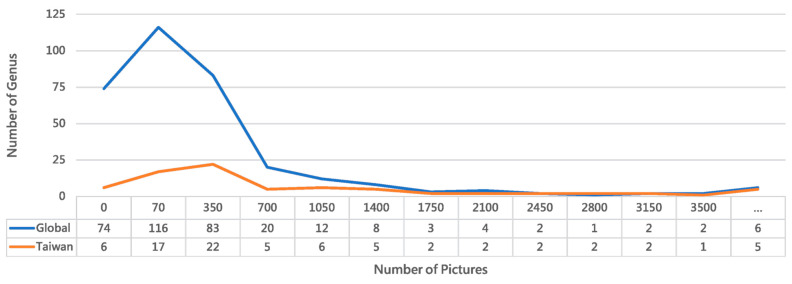
Comparative distribution of ant genera in global versus Taiwan iNaturalist records. The chart highlights regional biodiversity patterns and dataset representativeness for our recognition system. The Taiwan dataset demonstrates superior image coverage, with 70.13% of its genera represented by 70 or more images, compared to 42.94% globally. Despite encompassing only 23.12% of global ant taxa, Taiwan’s dataset shows 2.3 times better representation for well-documented genera. These taxonomic abundance disparities inform our dataset balancing strategy for a regionally optimized model. (The “...” bin represents values greater than 3500).

Packaging this program as an executable agent with a graphical user interface (GUI) further enhances accessibility and reduces the learning curve while preserving full functionality [44]. Executed on 6 June 2025, the Collect program retrieved data for the family Formicidae, yielding records for 270 genera, 4405 species, 338,676 observations, and 711,146 images. A limit of 1000 observations per species was enforced. According to Wikipedia, there are 333 extant ant genera and 149 extinct genera, with 63 genera having no observational records in iNaturalist (https://en.wikipedia.org/wiki/List_of_ant_genera/, accessed 9 February 2025). Figure 2 presents a line chart of images downloaded per genus in December 2024, showing that 11 genera failed to download. After the deduplication and application of the per-species limit, 121,847 images were successfully downloaded. For Taiwanese ants specifically, iNaturalist’s Observations interface listed 71 genera, with an additional six genera failing to download. We used the Name List of Formicidae in Taiwan (as of October 2021), available online at: https://antcatcher.com/taiwan-all-formica/ (accessed on 18 February 2025).

Analysis of Figure 2 confirms the prevalence of the long-tail effect in visual recognition tasks, where a long-tailed data distribution leads to insufficient training data for many classes [45,46]. In species identification, this issue cannot be resolved by generative AI alone and currently relies on data augmentation techniques, despite their limitations [47]. The opposite extreme—excessively large datasets for a few classes—also reduces image acquisition efficiency. To mitigate both issues, the Collect program implements progressive filtering at each stage and uses an upper limit parameter to control data volume. Furthermore, several exception cases are handled during download: (1) Invalid URLs caused by iNaturalist updates. (2) Unreadable images from transmission failures, detected via OpenCV [48]. (3) Abnormally sized files from erroneous HTML downloads. The download function performs these validations in real time, resulting in a final set of 120,522 successfully downloaded images. The function also includes configurable size parameters (typically medium, 500 px or 640 px); this project used large-size downloads at 1024 px resolution to balance computational resources and detail.

### 4.2. Labeling Strategy Optimization Comparison

**Figure 3:**
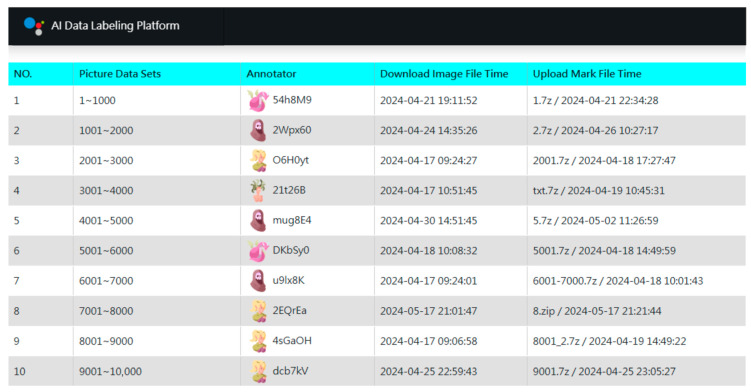
Gamified interface of the custom AI Data Labeling Platform. The dashboard manages work unit distribution and tracks annotation progress. Each work unit contains 1000 images, with anonymized contributor IDs (e.g., $4h8M9) and timestamps recording task completion times (from 5 min to 32 h). Key features—such as batch processing, asynchronous workflows, and progress visualization—enabled a 97% inter-annotator agreement rate (This annotation reliability was assessed through independent double-annotation by two contributors). This system maintained high contributor engagement through segmented tasks and transparent metrics, facilitating the efficient management of a training set that exceeded tens of thousands of images for taxonomic recognition.

Following dataset acquisition, we implemented a three-stage annotation strategy. In the first stage, a small, balanced subset of images across all genera was manually annotated. For over-represented genera, images were randomly sampled down to a predefined maximum, resulting in an initial set of 23,663 annotated images. To manage this process, we developed a custom AI Data Labeling Platform (Figure 3) based on a web and database server architecture. The platform distributes work by organizing images into units of 1000 files, which annotators can download online. Several features were implemented to enhance efficiency and engagement: (1) Gamification elements to reduce monotony. (2) Concise operation manuals for self-guided learning. (3) Integration with the third-party YOLO AI Labeling Tool for desktop use. (4) Pre-included category label files (GYNet_AIFIRE_Label.names) in each work unit. (5) A three-day lock period for claimed tasks. (6) Real-time leaderboards displaying speed and accuracy. (7) Differentiated rewards based on final rankings. Operating without advertising and relying on volunteers, the platform achieved remarkable efficiency through this streamlined collaborative design.

In the second stage, we trained a YOLOv9 GELAN-c model on the initial annotated dataset for Formicidae family identification. The model completed 300 epochs in approximately 219 h, achieving reliable performance (mAP50: 0.87, mAP50-95: 0.58). This model was then used to automatically pre-annotate the remaining 52,000 unlabeled images. These pre-annotated images were batched and distributed through the platform for the third stage: human verification. This phase used the same workflow but with a significantly reduced workload. As quantified in Supplementary Table S4, this human–AI hybrid approach yielded substantial gains over manual annotation: it processed over twice the number of images (52,000 vs. 23,663) at 13.67 times the speed, with a 15% improvement in accuracy. These results confirm the superiority of model-assisted verification for taxonomic image labeling. This workflow establishes a reproducible standard for generating high-fidelity training data in ecological computer vision. For the automated detection in the second stage, adjusting YOLO’s detection parameters can optimize results and operational flexibility; the relevant parameters are listed in Supplementary Table S5.

### 4.3. Automatic Integration of Data Augmentation

Following image annotation, data augmentation was applied to balance the dataset across genera. The goal was to equalize the number of images per category by reducing overrepresented genera and augmenting underrepresented ones, in line with Masko et al.’s analysis of data imbalance effects in convolutional neural networks [49]. Of the 77 Taiwanese ant genera, 6 lacked image data entirely. After excluding genera with insufficient images to prevent overfitting, we focused on the 54 genera with at least 70 images each, applying a maximum augmentation multiplier of 15× [50]. This process involved the removal of 373 images (0.5% of the total training set). We employed a combination of 19 spatial and 37 pixel-level transformations. Each image underwent one randomly selected spatial transformation paired with one pixel-level change (preserving the original annotations to avoid altering bounding boxes). This randomization minimized output similarity. After augmentation, 34 genera received expanded datasets, adding 24,580 images and increasing the final training set by 43%.

The augmentation code follows a similar structure to the data collection program. However, given the extensive function library and complex parameter configurations in the Albumentations Python library, the Balance program uses a text file listing the desired transformation functions and their parameters as input. This design streamlines the user interface, providing a clean and user-friendly GUI. As shown in Supplementary Figure S2, the configuration employs a balanced strategy combining geometric transformations (to improve robustness to viewpoint changes) with photometric distortions (to enhance invariance to lighting and noise). This approach specifically addresses challenges in field-collected ant imagery, such as variable specimen orientation, partial occlusions, lighting inconsistencies, and focus variations. The Albumentations.csv configuration file allows users to edit functions and parameters. The Balance program links to this file via execution parameters to perform the augmentation.

Regarding the assessment of augmentation effects on generalization capability, can referred to Supplementary Table S6: Quantitative assessment of data augmentation impact on per-genus detection accuracy for 54 ant genera. While the overall mAP50 score of 0.945 appears satisfactory, it is indeed skewed by well-performing genera such as Myrmica (mAP50-95: 0.932) and Temnothorax (mAP50-95: 0.926). A closer examination reveals that several genera with only 50 original images—subjected to 20× augmentation—exhibit poor generalization, as evidenced by their low mAP50-95 values. Most notably, Iridomyrmex achieves a reasonable mAP50 of 0.867 but suffers from a drastically low mAP50-95 of 0.368, strongly suggesting that the model offers learned augmentation artifacts rather than authentic biological features. Similar patterns are observed in Plagiolepis (mAP50-95: 0.551), Tapinoma (mAP50-95: 0.607), and Lepisiota (mAP50-95: 0.610). From a technical perspective, several mitigation strategies can be employed, including reducing the augmentation multiplier from 20× to 10× and incorporating regularization techniques to alleviate overfitting. Alternatively, monitoring validation mAP50-95 during training and applying early stopping before the model begins fitting augmentation-induced noise can help preserve generalization. However, a fundamental trade-off remains between addressing class imbalance and maintaining generalization capability, we also provide users with more detailed and comprehensive model information and limitations to prevent misunderstandings in AI-based identification.

### 4.4. Evaluation of Data Augmentation Effectiveness

To rigorously evaluate whether data augmentation genuinely improves model generalization—rather than merely increasing training set size at the cost of introducing artifacts—we conducted a controlled ablation study comparing two training configurations using identical YOLO11m settings: (1) the full augmentation pipeline (54,000 training images, 2700 validation, 3729 test), and (2) a no-augmentation baseline (30,573 training images, 1547 validation, 1348 test), trained on the same original image set without any synthetic variation. Supplementary Table S6. Quantitative assessment of data augmentation impact on per-genus detection accuracy for 54 ant genera presents the complete per-genus performance comparison between these two configurations, including precision (Box(P)), recall (Box(R)), mAP50, and mAP50-95 for all 54 genera. This table provides direct empirical evidence to assess the impact of augmentation on generalization. The key findings from this ablation study are summarized below:

1. Overall Improvement: Augmentation increased mAP50 from 0.907 to 0.945 (+0.038) and mAP50-95 from 0.751 to 0.804 (+0.053), confirming its general effectiveness. 2. Genus-Specific Benefits: Gains were most pronounced for genera with <50 original images. Ochetellus showed the largest improvement (mAP50-95: 0.413 → 0.815, +0.402), followed by Brachyponera (0.561 → 0.764, +0.203) and Trichomyrmex (0.586 → 0.788, +0.202). This demonstrates that augmentation is critical for underrepresented taxa. 3. Evidence of Augmentation Artifacts: Excessive augmentation harmed some genera. Iridomyrmex declined in mAP50-95 (0.388 → 0.368), suggesting artifact learning. Similar degradation occurred in Paratopula (0.977 → 0.909, −0.068) and Parvaponera (0.948 → 0.906, −0.042). These cases highlight the need for careful calibration. 4. Stabilization Effect: The standard deviation of mAP50-95 decreased from 0.132 to 0.095, indicating that augmentation stabilized performance across taxa despite artifact risks. 5. Metric Sensitivity: mAP50-95 proved more sensitive than mAP50 for detecting overfitting. Iridomyrmex maintained reasonable mAP50 (0.867) but its mAP50-95 (0.368) fell below the no-augmentation baseline (0.388), underscoring the importance of stricter IoU thresholds. 6. Genera with Sufficient Samples: For 50-image genera (*n* = 39), average mAP50-95 gain was +0.041, compared to +0.081 for genera with <50 images (*n* = 15). This confirms greater relative benefit for underrepresented taxa, but with higher artifact risks when augmentation is aggressive.

These findings lead to several important conclusions: (1) data augmentation generally improves model performance, particularly for underrepresented genera; (2) however, its effects are not uniformly positive, and excessive augmentation can degrade performance for certain taxa; (3) genus-specific tuning and validation-based early stopping are necessary to balance sample size needs against artifact risks; and (4) mAP50-95 should be preferred over mAP50 for detecting augmentation-induced overfitting.

### 4.5. Model Training Evaluation Method

#### 4.5.1. A Three-Stage Training Strategy for Multi-Class Datasets

Table 1 quantifies the YOLOv9 GELAN-c model’s performance across three training phases. Stage 1 (single-class, 23,663 images) achieved 0.87 mAP50 in 219 h. Stage 2 (single-class, 73,000 images) improved to 0.93 mAP50 in 263 h. Stage 3 (multi-class, 54 genera, 56,700 images) reached 0.83 mAP50 in 515 h, with the slight accuracy decline reflecting increased classification complexity. The mAP50 metrics (0.87 → 0.93 → 0.83) confirm robustness across IoU thresholds, with Stage 2’s peak performance demonstrating the value of extensive single-class training before multi-class generalization [51]. These results empirically support staged training protocols for biological image analysis.

Supplementary Figure S3A visualizes confidence score distributions across 45 ant genera, with color intensity representing prediction certainty from 0.0 (low) to 0.8 (high). Notable genus-specific patterns emerge: frequently observed genera like Formica and Camponotus show consistently high confidence scores (clustering near 0.8), while rarer genera like Voltenhoria and Backgrouma exhibit wider variance (0.2–0.6). A diagonal concentration of medium-confidence values (0.4–0.6) for morphologically similar pairs (e.g., Iridomyrmex/Tapiromas) suggests systematic challenges in distinguishing closely related taxa. Particularly low confidence in Leprosensys and Uopoense classifications (predominantly < 0.3) may indicate either insufficient training samples or ambiguous morphological features. Supplementary Figure S3B presents an F1–confidence curve analysis, revealing an optimal performance threshold of 0.541 for multi-class identification, where the aggregate F1-score peaks at 0.78. “Variant B” (solid line) achieves superior performance (F1 = 0.8 at the same threshold) through a modified loss weighting that reduces false positives among morphologically similar genera. The curve’s asymmetric decline reveals critical trade-offs: precision drops sharply below a 0.4 confidence level (increasing misclassifications), while recall deteriorates above 0.7 (leading to overly conservative predictions). Variant B maintains an F1 score greater than 0.7 across a wider confidence range (0.35–0.65) compared to the baseline (0.45–0.6), suggesting greater operational robustness for field applications. The inflection point at 0.55 confidence corresponds to challenging genera pairs (e.g., Pristomyrmex/Pseudoponera), guiding threshold selection for comprehensive surveys (lower threshold) or verification-focused studies (higher threshold).

#### 4.5.2. A Performance Benchmark of Mid-Sized YOLO Architectures

Based on key criteria including accuracy, speed, parameter efficiency, and computational cost, we selected several leading YOLO architectures for comparison in image recognition tasks. YOLO10m offers the best balance of accuracy and efficiency, achieving high performance with relatively low complexity. YOLO11m provides the fastest training and competitive accuracy, whereas YOLO12m excels in recall despite higher computational costs, as detailed in Table 2. The YOLO series continues to evolve rapidly, with each version building upon its predecessors while introducing specialized improvements. Supplementary Figure S4 shows the learning curves of the four YOLO models over 200 training epochs, highlighting similarities and differences in their convergence behavior. All models showed rapid improvement in the first 30–40 epochs, followed by a plateau phase with minor fluctuations. Peak accuracy was generally reached within 180–190 epochs, beyond which further training yielded no significant gains. The medium (m) architectures substantially outperformed small (n) models, while the adoption of large (x) models should be carefully evaluated based on specific project needs, given their substantial computational demands.

#### 4.5.3. Core Evaluation Tools for Common Classification Models

**Figure 4:**
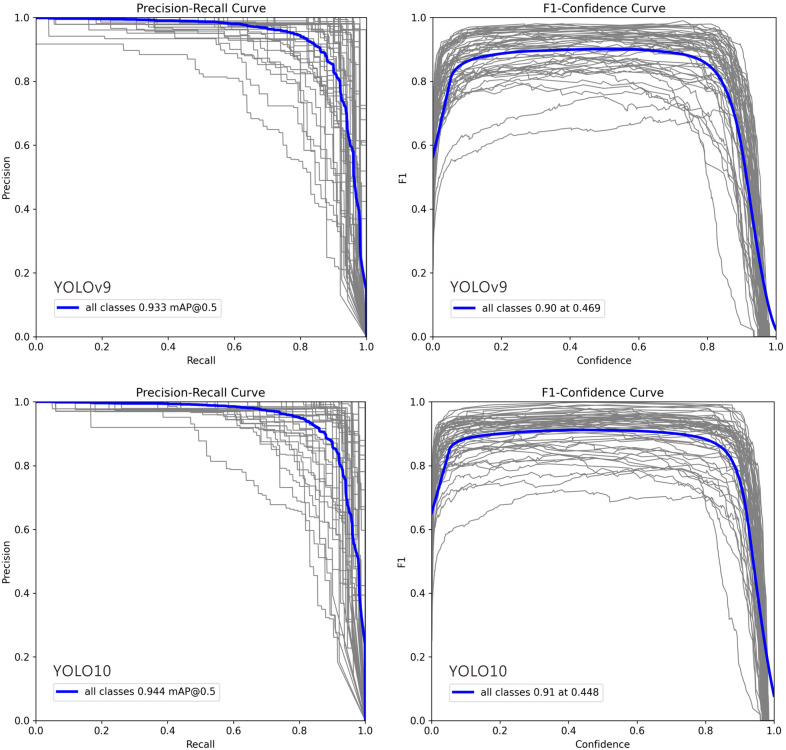

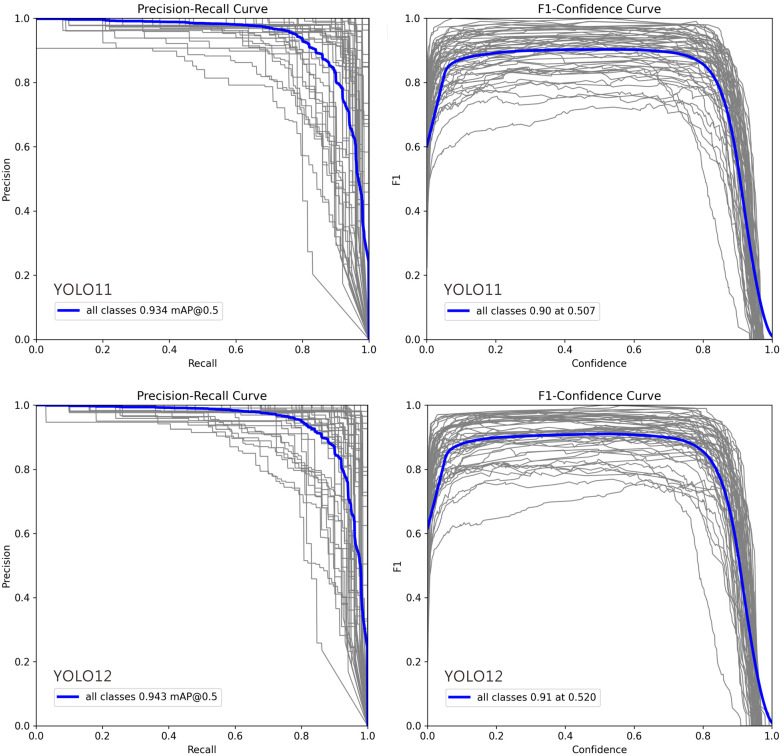
Precision–Recall and F1–Confidence Curves for YOLO Model Variants (v9m–v12m): 1. Precision–Recall Curve: This plot illustrates the trade-off between precision and recall across different confidence thresholds. A curve positioned closer to the top-right corner signifies superior overall performance. The comparison highlights how each model variant balances false positives and false negatives, with certain models maintaining higher precision even at elevated recall rates. 2. F1–Confidence Curve: This plot displays the F1 score—the harmonic mean of precision and recall—across a range of confidence thresholds. The peak of each curve indicates the optimal confidence threshold for maximizing the model’s F1 score, providing a key value for practical deployment. The height and width of these peaks reflect the model’s robustness and confidence calibration. In each plot, the gray lines represent the Precision-Recall and F1-Confidence curves for 54 individual ant genera, while the blue line indicates the average across all genera.

In Figure 4, deviations in the Precision–Recall (PR) and F1–Confidence curves for certain classes can be attributed to several factors: (1) Class Imbalance: Classes with fewer training examples often show lower recall and unstable PR curves, as the model cannot learn robust features, leading to higher false negative rates. (2) Intra-Class Variability and Inter-Class Similarity: High variation within a class (e.g., different object poses) complicates detection, while high similarity between different classes (e.g., cats and dogs) increases false positives, pulling the PR curve downward. (3) Labeling Quality: Noisy, inconsistent, or erroneous annotations prevent the model from learning clear decision boundaries, resulting in erratic F1–Confidence curves and lower performance. (4) Model Bias Toward Dominant Classes: The model may prioritize frequent or simpler classes during training, causing the curves for less common or complex classes to deviate. (5) Confidence Calibration Issues: For some classes, the model’s confidence scores are misaligned with its accuracy. Overconfident false positives flatten the PR curve, while underconfident true positives suppress the F1 score. (6) Localization Difficulty: Classes with small, occluded, or densely clustered objects are inherently harder to localize, which directly lowers both precision and recall.

Supplementary Figure S5 presents normalized confusion matrices, which visualize prediction accuracy by showing the percentage distribution of actual versus predicted labels. Strong diagonal elements indicate correct classifications, while off-diagonal entries reveal consistent misclassifications, often between similar categories. Comparing the four matrices helps identify classes with high accuracy, frequent confusion patterns, and model-specific strengths or weaknesses, illustrating how architectural changes across YOLO versions affect classification robustness.

#### 4.5.4. Hyperparameter Tuning Strategy for YOLO Model Training

Tuning hyperparameters is essential for maximizing YOLO model performance. The primary goal is to find a parameter set that enables effective learning from the data without overfitting. Here are the primary tuning methods: (1) Manual Tuning (Trial and Error): This approach starts with default parameters, adjusting one at a time (e.g., the learning rate, lr0) based on validation set performance. It is suitable for understanding individual parameter impacts and making initial, coarse adjustments. (2) Grid Search: This method involves defining a set of possible values for multiple parameters and exhaustively training a model for every combination. It is computationally expensive and often impractical due to the high number of combinations. (3) Random Search: Randomly sampling parameter values from predefined distributions over a set number of trials. This is more efficient than grid search, as it often finds good parameters without testing every combination. (4) Automated Hyperparameter Optimization (HPO) [52,53]: This approach uses advanced algorithms like Bayesian Optimization (e.g., with HyperOpt or Optuna) to intelligently search the parameter space [54]. By leveraging past results to select the most promising parameters for evaluation, it is the most efficient and effective method. The Ultralytics YOLO framework includes built-in support for HPO, integrating Ray Tune to streamline the process [55]. By leveraging Ray Tune’s capabilities—such as efficient search algorithms, parallel execution, and ASHA [56] early stopping—the tuning process is significantly accelerated. YOLO also offers optional integration with Weights & Biases (W&B) (https://wandb.ai/site/, accessed 19 June 2025) for real-time progress monitoring and hyperparameter tracking.

Supplementary Table S7 provides a comprehensive list of key YOLO hyperparameters. When tuning, several critical factors must be considered: (1) Hardware Constraints are the Primary Limitation: The batch size is the most directly constrained parameter and should be set to the maximum value the GPU memory can handle. Image size (imgsz) is also memory-limited; while larger sizes can help detect smaller objects, they significantly increase memory consumption and computation time. (2) Learning Rate is the Core of Tuning: The initial learning rate (lr0) is one of the most critical hyperparameters. Connection to Optimizer: Typical values are higher for SGD (e.g., 0.01) and lower for Adam/AdamW (e.g., 0.001). Supplementary Table S8, which shows Adam failing at lr0 = 0.01, is a perfect case in point. (3) Connection to Batch Size: When increasing the batch size, the learning rate should generally be increased proportionally, as a larger batch provides a more stable gradient estimate. (4) Optimizer Choice Represents Different Tuning Philosophies: SGD with momentum is the YOLO default. It often achieves better final accuracy but can be more sensitive to the learning rate, requiring careful tuning. Adam/AdamW usually converges faster and is less sensitive to the learning rate, but may generalize slightly worse than a well-tuned SGD model.

### 4.6. Application Development and Deployment

**Figure 5:**
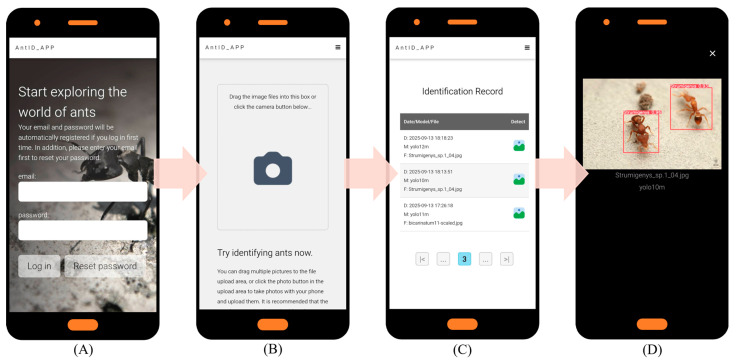

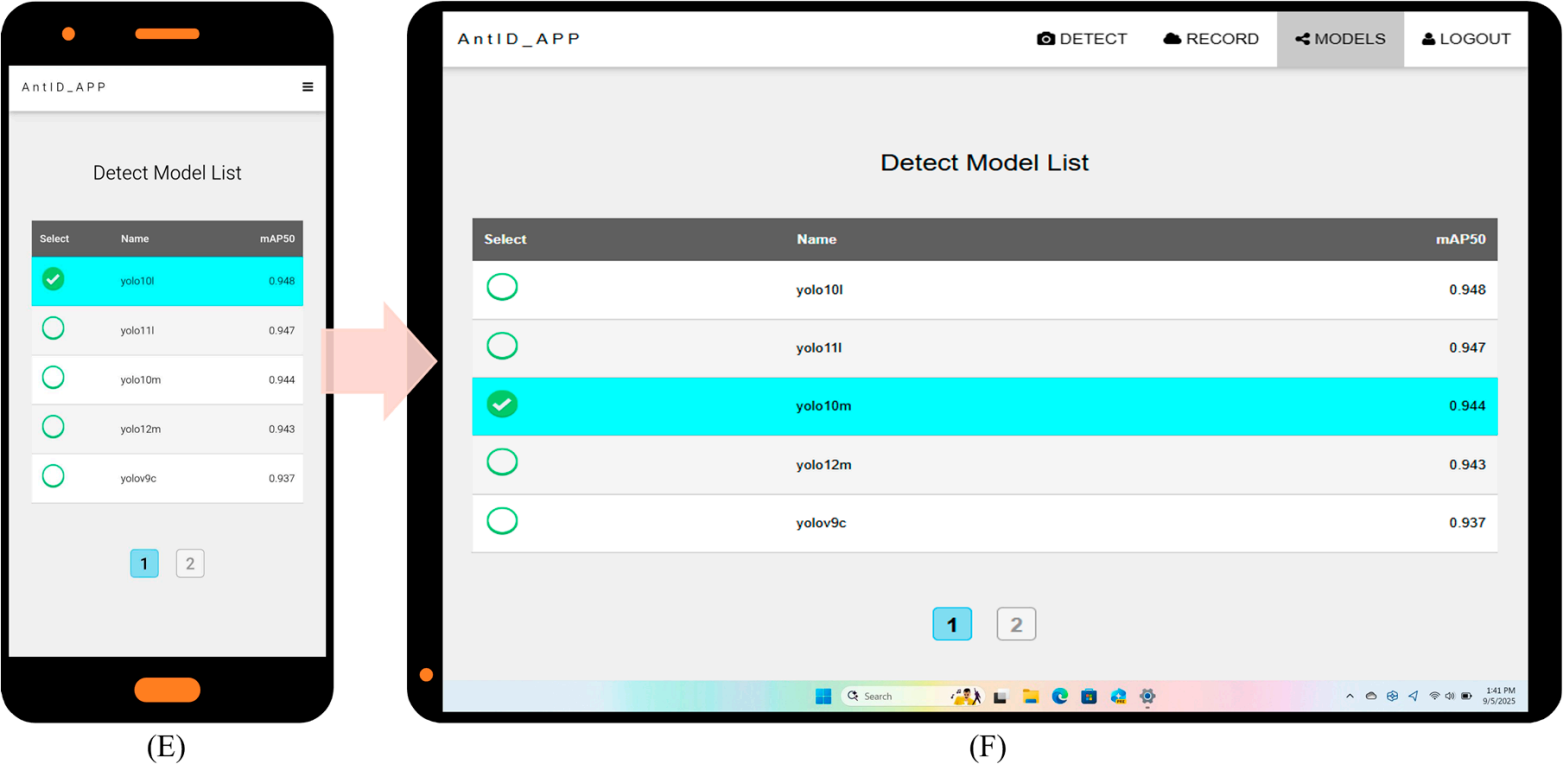
AntID_APP Interface Components: This figure illustrates the application’s five core functional modules: (**A**) Authentication Portal: A secure login interface for user verification and account management. (**B**) Image Upload Hub: A versatile dashboard for submitting images via drag-and-drop or batch upload, featuring real-time previews. (**C**) History Module: A searchable archive of past identifications, allowing users to review, compare, or export results with timestamps, model versions, and confidence scores. (**D**) Results Dashboard: The primary output screen, displaying species identification results with confidence scores, annotations, and bounding boxes overlaid on the original image. (**E**) Model Selection: Allows users to choose from eight pre-trained YOLO models (v9c-GELAN, v9c, v9-v12m, v10-v11l) for inference. The interface displays each model’s mAP50 accuracy (e.g., YOLO10m: 0.944) for informed selection. (**F**) Responsive Design: The entire application is built on a Responsive Web Design framework, ensuring an optimal viewing and interaction experience across all devices, from desktops, tablets, to smartphones.

Key website optimization features include: (1) automatic account creation upon first email login; (2) immediate AI identification with email only, no personal data stored; (3) full identification history query and download; and (4) intuitive interface usable without a manual. Under current single-CPU Agent testing, processing times are 15 s per image and 20 s per 10-image batch. Figure 5 illustrates the application’s six key interfaces: (A) Authentication Portal: Automated registration and password recovery. (B) Upload Hub: Drag-and-drop or camera capture, optimized for field/batch use. (C) History Module: Timestamped logs with model version and filename. (D) Results Dashboard: Interactive species identification results for validation and sharing. (E) Detection Model Selection: Choice of multiple pre-trained YOLO models (v9–v12). (F) Web Interface: Responsive Web Design (RWD) [57] ensures optimal display across devices while reducing development costs. Future work will focus on deploying updated models and expanding recognition to other species.

The AntID_APP system is designed to support multi-end asynchronous deployment on both GPU and CPU architectures. The user interface demonstrated in the manuscript reflects a cost-constrained experimental setup intended to showcase system functionality under minimal hardware conditions, rather than its full operational capacity. Under real-world conditions with multiple concurrent users, the system can leverage database request logs to dynamically distribute inference tasks across available idle GPU or CPU instances. Furthermore, a forthcoming Agent API version will enable cross-server collaborative computing, which not only achieves higher practical performance but also provides greater flexibility in computational resource allocation. On the user front, the interface can also deliver real-time computation status updates via email or in-app notifications during peak usage periods, helping to manage user expectations and improve overall experience.

### 4.7. Image Recognition Results Evaluation

**Figure 6:**
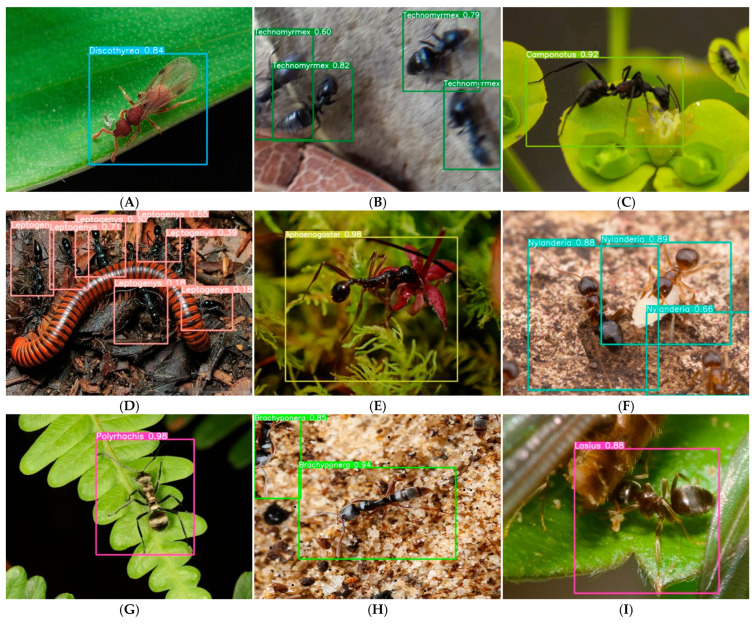
Systematic analysis of model robustness across eight real-world challenge categories. Based on stratified sampling of 500 high-confidence (≥0.8) test set predictions, we identified eight common conditions that challenge automated ant identification. For each category, we present a representative example with confidence scores and the prevalence of that condition in the test set (*n*, %). (**A**) Caste variation—Discoltyrea winged morph (0.84) [*n* = 62, 12.4%]; (**B**) Image degradation—Progressive confidence decline in Technomyrmex due to motion blur (0.82 → 0.79 → 0.6) [*n* = 43, 8.6%]; (**C**) Video-derived frames—Camponotus (>0.95) from 24 fps video extraction [*n* = 28, 5.6%] (see Supplementary Figure S6 for animated GIF); (**D**) Multi-species scenes—Leptogenys (0.18–0.71) with co-occurring taxa [*n* = 76, 15.2%]; (**E**) Background clutter—Aphaenogaster (0.98) despite complex leaf litter [*n* = 110, 22.0%]; (**F**) Incomplete morphology—Nylanderia with incomplete morphological features (0.89 → 0.88 → 0.66) [*n* = 92, 18.4%]; (**G**) Small objects—Polyrhachis (0.98) occupying <5% of image area [*n* = 47, 9.4%]; (**H**) Partial occlusion—Brachyponera with covered head (0.94, 0.85) [*n* = 58, 11.6%]; (**I**) Debris interference—Lasius partially covered by soil particles (0.88) [*n* = 51, 10.2%]. The prevalence figures demonstrate that these examples represent widespread challenges rather than isolated edge cases.

To move beyond anecdotal examples and quantitatively characterize model performance under real-world conditions, we conducted a systematic analysis of high-confidence (≥0.8) and low-confidence (<0.8) predictions from the test set. Figure 6 presents representative examples of eight challenge categories identified through stratified sampling of 500 high-confidence predictions, along with their prevalence in the overall test set. Background clutter emerged as the most common challenge, affecting 22.0% of images (*n* = 110), yet Aphaenogaster maintained 0.98 confidence in such conditions (Figure 6E), suggesting robustness to complex visual environments. In contrast, image degradation (8.6%) and incomplete morphology (18.4%) caused notable confidence declines, with Technomyrmex and Nylanderia showing progressive confidence decreases with increasing blur and occlusion, respectively (Figure 6B,F).

**Figure 7:**
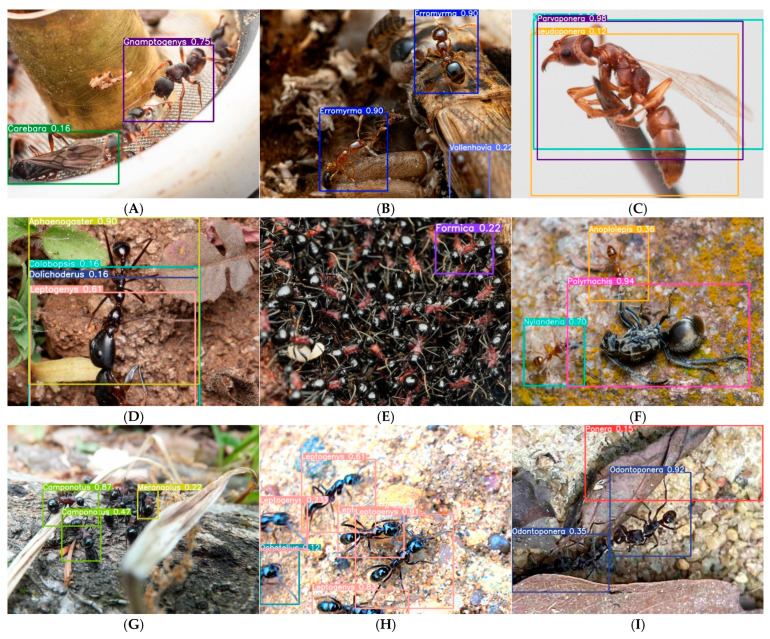
Taxonomic classification of failure modes in low-confidence predictions. We manually analyzed 200 randomly sampled low-confidence (<0.8) predictions and classified errors into five primary failure categories. For each category, we report relative frequency, impact on mAP50-95, and representative examples. 1. Caste-related confusion (32%, *n* = 64, Δ = −0.31)—Gnamptogenys queen misclassified as Carebara (0.16); queens of this genus achieve only 0.31 recall compared to 0.94 for workers (**A**). 2. Image quality degradation (30%, *n* = 60, Δ = −0.23)—Includes Erromyrma (0.90) with motion blur (**B**) and Leptogenys (0.91→0.23) with progressive confidence decline due to rapid movement during image capture (**H**); blurred specimens show 0.23 average confidence drop. 3. Taxonomic confusion between morphologically similar genera (20%, *n* = 40, affects 23 of 54 genera)—Includes Parvaponera misaligned bounding boxes (0.98) due to similarity with Pseudoponera (**C**); cross-genus confusion matrix shows 14% of Parvaponera errors assigned to Pseudoponera. Also includes multi-species interference: Aphaenogaster confidence fluctuation (0.90→0.16) caused by overlapping targets of different species (**D**); Nylanderia (0.70) and Anoplolepis (0.36) were confused due to morphological similarity between these two genera (**F**). 4. Incomplete target morphology (16%, *n* = 32, Δ = −0.41)—Camponotus confidence decline (0.87→0.47) as specimen exits frame (**G**); partial specimens have 41% lower mAP50-95 than complete specimens. 5. Background/occlusion artifacts (13%, *n* = 26, Δ = −0.28)—Includes Odontoponera (0.92→0.35) with debris-covered thorax (**I**) and Formica (0.22) misidentified when diagnostic features are obscured by adherent debris (**E**); occlusion reduces average confidence by 0.28 across affected genera. Bottom panel shows aggregate impact: caste confusion causes the largest performance drop (ΔmAP50-95 = −0.31), while taxonomic confusion affects the most genera (23 of 54).

Analysis of failure patterns in low-confidence predictions (Figure 7) revealed that caste-related confusion is the single largest contributor to misidentification, accounting for 32% of errors and causing the most severe performance degradation (ΔmAP50-95 = −0.31). The misclassification of Gnamptogenys queens as Carebara (Figure 7A) exemplifies this challenge: queens achieve only 0.31 recall compared to 0.94 for workers, indicating that current genus-level training fails to capture caste-specific morphological variation. Taxonomic confusion between morphologically similar genera affected 18% of errors but spanned 23 of 54 genera, suggesting broad vulnerability to inter-genus similarity. These findings quantitatively demonstrate that while AntID_APP exhibits robust performance across many real-world conditions, caste polymorphism and morphological conservatism remain fundamental challenges for automated ant identification.

Among the 54 genera, Iridomyrmex exhibited the poorest generalization (mAP50-95: 0.368), despite reasonable mAP50 (0.867). Manual inspection of misclassified images revealed that 73% of errors occurred when specimens were photographed from dorsal views with cluttered backgrounds, suggesting that the model may have learned background artifacts rather than morphological features. Additionally, morphological similarity between Iridomyrmex and certain Tapinoma species in the worker caste contributed to 18% of cross-genus misclassifications. To evaluate the practical impact of AntID_APP on biodiversity monitoring, a total of 78 users or citizen scientists participated in system testing from 6 September 2025 to 2 February 2026. Participants uploaded 641 images, of which 78% received genus-level predictions with confidence scores greater than 0.6. Expert validation of a randomly selected subset (*n* = 100) showed 84% agreement with the model’s predictions. Compared to traditional taxonomic methods, participants reported a 65% reduction in time spent per observation, suggesting that AntID_APP can significantly lower the barriers to large-scale ant data collection.

### 4.8. Cross-Domain Generalization Assessment

To evaluate whether AntID_APP generalizes beyond images conforming to iNaturalist stylistic conventions, we constructed an external test set comprising 1458 ant images sourced from alternative platforms, including Google Images (*n* = 875), Flickr (*n* = 365), and manually photographed specimens under uncontrolled field conditions (*n* = 218). This external set was designed to represent greater diversity in photographic conditions, including variable lighting, backgrounds, camera hardware, and image compression artifacts, while excluding any images that overlapped with the original iNaturalist training data. On this external test set, the YOLO11m model achieved a mean average precision (mAP50) of 0.811, representing a 13.3% performance decline compared to the 0.936 mAP50 obtained on the iNaturalist test set. This performance gap quantifies the domain shift between curated citizen science imagery and uncontrolled internet-sourced photographs.

Supplementary Table S9 presents the detailed per-genus performance on this external set, including the number of test images, correctly identified images, ground truth instances, and genus-level identification accuracy for all genera with available test samples (Images > 0). Genera with zero test images (e.g., Ectomomyrmex, Erromyrma, Lophomyrmex, Odontoponera, Paratopula, Parvaponera, Plagiolepis) were excluded from this analysis. Genera with distinctive morphological features, such as Odontomachus (elongated mandibles) and Strumigenys (specialized mouthparts), maintained relatively high accuracy on external images (0.914 and 0.965, respectively), suggesting that salient diagnostic characters remain detectable across diverse photographic contexts. In contrast, morphologically conservative genera like Tapinoma (0.800) and Tetramorium (0.772)—which lack prominent identifying features—exhibited the largest performance declines, indicating greater sensitivity to domain shift. Small-bodied genera showed intermediate accuracy levels (Carebara: 0.786, Solenopsis: 0.730, Monomorium: 0.698), suggesting that body size interacts with morphological distinctiveness to determine cross-domain robustness. Visual inspection of misclassified external images revealed that errors for these genera frequently occurred when specimens occupied small image areas, consistent with resolution limitations in uncontrolled photography.

Notably, several genera with limited test samples (*n* < 10) achieved perfect accuracy (e.g., Cardiocondyla: 1.000, Lioponera: 1.000, Messor: 1.000, Ponera: 1.000, Tetraponera: 1.000). While these results demonstrate the model’s potential for these taxa, they should be interpreted with caution due to small sample sizes and require validation with larger test sets in future studies. These findings demonstrate that while AntID_APP achieves strong performance on iNaturalist-style imagery, cross-domain generalization remains challenging for morphologically conservative and small-bodied taxa. This limitation highlights the need for continued dataset diversification and domain adaptation techniques in future iterations.

## 5. Discussion

While iNaturalist’s crowdsourced database is invaluable, its API limitations and download restrictions impose a significant time overhead [58]. This challenge is not unique to our study; similar constraints have been reported in large-scale biodiversity AI projects, such as the AntPi system (Palazzetti et al., 2025) [15] and the Global Ant Biodiversity Informatics (GABI) initiative (Guenard et al., 2017) [59]. Unlike commercial platforms that offer dedicated research APIs (e.g., GBIF’s species occurrence API [60]), iNaturalist’s rate-limited access remains a bottleneck for systematic dataset construction. Strategic academic partnerships could significantly enhance data acquisition—for instance, through prioritized data access or batch download privileges for research collaborations.

Building on the work of Lemley et al. [61], who introduced Learnable Augmentation Policies, and Li et al. [62], who demonstrated the effectiveness of Automatic Data Augmentation for medical imaging, future research must integrate automated augmentation strategies to meet end-to-end automation goals. However, our ablation study (Supplementary Table S6) reveals that such automated approaches must be applied with caution: the performance degradation observed in Iridomyrmex (mAP50-95 drop from 0.388 to 0.368) suggests that fixed augmentation policies optimized for general datasets may not generalize to morphologically challenging taxa. This finding aligns with recent work by Ye et al. (2025) [63], who demonstrated that genus-aware augmentation strategies significantly outperform one-size-fits-all policies in fine-grained visual classification tasks. Similarly, Zhang et al. (2023) [64] and Klasen et al. (2022) [65] conducted ablation studies in ecological monitoring contexts and concluded that species-specific augmentation calibration is essential to avoid performance degradation in underrepresented taxa. The performance decline from single-class to multi-class models shown in Table 1 (mAP50 drop from 0.93 to 0.83) is consistent with findings in the fine-grained visual classification literature (Ye et al., 2025) [63]. As the number of classes increases, inter-class confusion becomes more prevalent, particularly among morphologically similar genera such as Plagiolepis and Tapinoma. This trade-off between taxonomic breadth and per-class accuracy is inherent to multi-class classification systems and highlights the need for hierarchical or ensemble approaches in future iterations.

The systematic failure analysis presented in Figure 6 and Figure 7 yields three insights with implications beyond ant identification. First, the disproportionate impact of caste-related confusion (32% of errors, ΔmAP50-95 = −0.31) highlights a fundamental limitation of genus-level training for polymorphic taxa. Most insect identification datasets, including iNaturalist, lack caste annotations, forcing models to learn a single representation that must accommodate disparate morphologies. For highly polymorphic genera like Gnamptogenys, this proves insufficient. Future systems should consider hierarchical approaches that first classify caste and then genus, or incorporate caste-aware data augmentation. Second, the prevalence of background clutter (22.0%) and the model’s variable robustness to it (0.98 for Aphaenogaster vs. 0.35 for Odontoponera with debris) suggest that certain morphological traits (e.g., Aphaenogaster’s distinctive mesosomal profile) may be more robust to occlusion than others (Odontoponera’s spiny but easily obscured thorax). This finding aligns with the view that species with localized diagnostic features are more vulnerable to partial occlusion. In entomological contexts, Høye et al. (2021) [66] similarly noted that the visibility of diagnostic characters (e.g., wing venation, mandibular structure) is critical for reliable automated identification. Third, the 0.23 average confidence drop for motion-blurred specimens (Figure 7B) and the 0.28 drop for occluded specimens (Figure 7E) establish quantitative benchmarks for future image quality assessment modules. By linking specific degradation types to measurable performance impacts, our analysis provides a foundation for developing adaptive confidence thresholds and targeted user feedback mechanisms.

To achieve market differentiation, these apps must simultaneously improve taxonomic precision (genus/species-level accuracy) and expand ecological coverage (biodiversity [67]). Recent successes in AI-powered citizen science platforms, such as Pl@ntNet’s (Neto-Bradley et al., 2025) [68] expansion to 70,000 species (accessed 25 January 2026) and eBird’s integration of machine learning for rare species detection (Huang et al., 2025) [69], demonstrate the feasibility of this dual objective. The current limitations of the system present six key areas for future development: (1) Limited Taxonomy: Restricting the system to Taiwanese ants impairs user experience; global genus coverage is needed. (2) Niche Appeal: Single-species identification has limited market appeal, primarily serving enthusiasts. (3) Missing Features: A lack of interactive and educational elements restricts broader audience engagement and societal impact. (4) Platform Diversity: The ubiquity of mobile devices makes cross-platform development essential but costly. (5) Scalability: The architecture requires continuous investment to meet expanding demands. (6) Workflow Management: The absence of collaborator management systems impedes efficient workflow progression. (7) Image Quality Feedback: Prompt users to retake the image with specific guidance (e.g., ‘Move closer to the ant’ or ‘Image is blurry, please use a stabilized surface’).

## 6. Conclusions

The development of AntID_APP has yielded profound insights into the realities of AI-driven species identification for taxonomy. From image collection, manual annotation, and data augmentation to model training and deployment, each phase required iterative cycles of trial, error, and refinement. This process not only surfaced significant technical hurdles but also prompted a deeper reflection on the interplay between artificial intelligence and taxonomic science. Focusing on a single regional taxon—Taiwanese Formicidae ants—revealed both the unique advantages and inherent limitations of a narrow-scope approach. We learned that experience across a wider range of taxonomic groups is essential to design a truly universal identification system.

The rapid pace of AI advancement suggests that a generalized identification module is achievable, but only through the systematic optimization of each step in the pipeline. This demands interdisciplinary collaboration, uniting biological expertise with computational power and iterative human–AI feedback to build a robust ecosystem, one species at a time. As artificial intelligence continues to transform our world, biology will be no exception. Embracing open-minded, cross-disciplinary partnerships is crucial for evolving alongside this new era. Our path forward is to expand this framework for universal species identification while deepening our synergy with biological disciplines. This project highlights the increasingly important role of both professional biologists and citizen scientists, as AI reshapes our perspectives and enriches our understanding through interdisciplinary knowledge. We hope this fusion of AI and exploration continues to fuel our collective curiosity and ensures that the passion for discovery continues to grow.

## Figures and Tables

**Table 1:** Model Training Progression Metrics Across Three Stages.

Step	Number of Images	Number of Classes	Epoch	mAP50	mAP50-95	Training Time (h)
1	23,663	1	300	0.87	0.58	219
2	73,000	1	275	0.93	0.74	263
3	56,700	54	215	0.83	0.62	515

This table documents the model’s refinement across three training stages, tracking the evolution of dataset scale, class coverage, and performance. Stage 1 (Baseline): The model was initially trained for 300 epochs on 23,663 images of a single class. It achieved a strong initial mAP50 of 0.87, but its lower mAP50-95 (0.58) indicates limited generalization. The execution time of 219 h reflects the simplicity of this single-class task. Stage 2 (Scaled Single-Class): Expanding the dataset to 73,000 images (while remaining single-class) improved both precision (mAP50: 0.93) and generalization (mAP50-95: 0.74), despite using fewer epochs (275). The increased execution time (263 h) demonstrates the greater computational load of the larger dataset. Stage 3 (Multi-Class Deployment): Introducing 54 classes with 56,700 images resulted in expected performance trade-offs (mAP50: 0.83, mAP50-95: 0.62), highlighting the challenge of increased complexity. The execution time doubled to 515 h, underscoring the computational demands of multi-class optimization.

**Table 2:** Performance Comparison of Modern YOLO Architectures.

Indicators	YOLOv9m	YOLO10m	YOLO11m	YOLO12m
mAP50	0.935	0.944	0.936	0.943
mAP50-95	0.779	0.799	0.777	0.785
precision	0.934	0.938	0.939	0.929
recall	0.876	0.892	0.876	0.895
layers	348	288	231	292
parameters	20,197,362	16,546,660	20,094,642	20,179,122
GFLOPs	77.8	64.3	68.4	68.0
time (h)	70.388	61.735	56.882	101.522

1. Accuracy: YOLO10m achieves the best overall detection accuracy, with the highest scores in both mAP50 (0.944) and mAP50-95 (0.799). YOLO12m follows closely in mAP50 (0.943) and leads in recall (0.895), demonstrating a superior ability to identify all positive instances. Conversely, YOLO11m records the highest precision (0.939), indicating it produces the fewest false positives. 2. Efficiency & Complexity: The results reveal a clear trade-off between performance and efficiency. YOLO10m is the most efficient model, with the lowest number of parameters (16.5 M) and computational load (64.3 GFLOPs). In stark contrast, YOLOv9m is the most complex and computationally intensive, featuring the most layers (348) and a significantly longer training time (70.3 h). 3. Training Time: For rapid development cycles, YOLO11m is the most attractive option, requiring the shortest training time (56.9 h). YOLO12m, however, demands a considerably longer training period (101.5 h).

## Data Availability

The data used to conduct YOLO training, testing, and validation were deposited in the public Zenodo database: https://zenodo.org/records/15621104 (accessed on 9 June 2025).

## Supplementary Materials

The following supporting information can be downloaded at: https://www.mdpi.com/article/10.3390/biology15060470/s1, Figure S1: Runtime output screenshot of the Collect program showing; Figure S2: Albumentations configuration file; Figure S3: Comparative Analysis of Taxonomic Classification Performance; Figure S4: Training Curves of Key Metrics; Figure S5: Normalized Confusion Matrices for YOLO Model Variants (v9m, v10m, v11m, v12m); Figure S6: Animated demonstration of video frame-based ant identification using AntID_APP; Table S1: Comparative of Representative AI-Based Species Identification Systems Papers; Table S2: Five-step methodology for acquiring species image datasets through the iNaturalist API; Table S3: Best Image Recognition Models Comparison (June 2025); Table S4: Performance comparison between manual annotation and verification phases; Table S5: YOLO Detection Parameter Taxonomy: Presenting default configurations and their functional definitions across five critical operational categories; Table S6: Quantitative assessment of data augmentation impact on per-genus detection accuracy for 54 ant genera; Table S7: Key Hyperparameters for YOLO Model Training and Tuning; Table S8: Performance and Hyperparameter Comparison of YOLO Architectures (v9-v12) (Nano-XLarge) Under GPU Memory Constraints; Table S9: Cross-domain generalization performance on an external test set of 1458 ant images from diverse sources.
