# Supplementary material for: AntID_APP: Empowering Citizen Scientists with YOLO Models for Ant Identification in Taiwan
Source: Biology (Basel). 2026 Mar 14;15(6):470. doi: 10.3390/biology15060470 (PMC13024282; doi:10.3390/biology15060470)
Supplement: Supplementary file 1 [file biology-15-00470-s001.zip › biology-4139908-supplementary.pdf]

Supplementary Material for AntID_APP: Empowering Citizen Scientists with YOLO Models for Ant Identification in Taiwan

Nan-Yuan Hsiung [1], Jen-Shin Hong [1], Shiu-Wu Chau [2] and Chung-Der Hsiao [3,*]

[1] Department of Computer Science and Information Engineering, National Chi Nan University, Nantou 545301, Taiwan; s110321901@ncnu.edu.tw (N.-Y.H.); jshong@ncnu.edu.tw (J.-S.H.)
[2] Department of Engineering Science and Ocean Engineering, National Taiwan University, Taipei 106319, Taiwan; chausw@ntu.edu.tw
[3] Department of Bioscience Technology, Chung Yuan Christian University, Taoyuan 320314, Taiwan
* Correspondence: cdhsiao@cycu.edu.tw

Contents of This File

Introduction

Table S1 through S9

Figure S1 through S6

Introduction

This supplementary document provides additional technical details, experimental results, and implementation specifications supporting the main manuscript "AntID_APP: Empowering Citizen Scientists with YOLO Models for Ant Identification in Taiwan". The primary study introduces AntID_APP, a practical system designed for accurate and efficient Taiwan ant genus recognition in field conditions using state-of-the-art YOLO (You Only Look Once) object detection models for Citizen Scientists. These resources are intended to assist researchers and developers in replicating the system or adapting the framework for related ecological monitoring or fine-grained object recognition tasks. The modular pipeline developed in this study can be seamlessly extended to AI recognition tasks for other species, thereby enabling the construction of a comprehensive multi-species collaborative detection system.

Table S1. Comparative of Representative AI-Based Species Identification Systems Papers.

Author	Year	System	Target Taxa	Geographic	Paper
**General Platforms**					
Joly et al.	2016	Pl@ntNet	Plants	Global	A look inside the Pl@ntNet experience: The good, the bias and the hope.
Hogeweg et al.	2024	iNaturalist	Multi-taxa	Global	AI Species Identification Using Image and Sound Recognition for Citizen Science, Collection Management and Biomonitoring: From Training Pipeline to Large-Scale Models.
Sullivan et al.	2009	eBird	Birds	Global	eBird: A citizen-based bird observation network in the biological sciences.
Picek et al.	2025	LifeCLEF	Multi-taxa	Global	Overview of lifeclef 2025: Challenges on species presence prediction and identification, and individual animal identification.
**General Insect ID**					
Sadia et al.	2025	AInsectID	General insects	Global	AInsectID Version 1.1: An insect species identification software based on the transfer learning of deep convolutional neural networks.
Chiranjeevi et al.	2025	InsectNet	General insects	Global	InsectNet: Real-time identification of insects using an end-to-end machine learning pipeline.
Gao et al.	2024	-	Insects	Global	Application of machine learning in automatic image identification of insects-a review.
Ejaz et al.	2025	-	Crop pests	Global	Crop pest classification using deep learning techniques: a review.
Lakyiere et al.	2025	-	Mosquitoes	Global	Trends and advances in image-based mosquito identification and classification using machine learning models: A systematic review.
**Specialized Arthropod ID**					
Luong et al.	2023	SpiderID_APP	Spiders	Taiwan	SpiderID_APP: A User-Friendly APP for Spider Identification in Taiwan Using YOLO-Based Deep Learning Models.

Palazzetti et al.	2025	AntPi	Ants (6 classes)	Europe	AntPi: A Raspberry Pi based edge-cloud system for real-time ant species detection using YOLO.
Apeinans et al.	2024	-	Ants	General	Ant Detection using YOLOv8: Evaluation of Dataset Transfer Impact.
Sorbellii et al.	2023	-	Halyomorpha halys	Orchard	A drone-based automated Halyomorpha halys scouting: A case study on orchard monitoring.
Zhang et al.	2026	-	Ant nests	Dikes	Research on an Improved YOLOv7-based Intelligent Ant Nest Recognition Method for Dam Drone Images.
Shaowei et al.	2026	-	Rice disease	Crops	LGH-YOLOv12n: Latent Diffusion Inpainting Data Augmentation and Improved YOLOv12n Model for Rice Leaf Disease Detection.
**Acoustic ID**					
Rowley et al.	2020	FrogID	Frogs	Australia	The FrogID dataset: expert-validated occurrence records of Australia's frogs collected by citizen scientists.
Lapp et al.	2021	-	Frogs	General	Automated detection of frog calls and choruses by pulse repetition rate.
**Bias & Methodology**					
Santoro et al.	2025	-	Mammals	General	Essential tools but overlooked bias: Artificial intelligence and citizen science classification affect camera trap data.

Presents a comparative overview of 18 representative AI-based species identification systems paper, organized into five thematic categories: general citizen science platforms, general insect identification systems, specialized arthropod identification tools (YOLO-based ecological applications), acoustic identification systems, and methodological studies on bias and evaluation. For each paper, we summarize the author, year, system, target taxa, geographic focus and paper title. This comparison highlights the diversity of approaches in the field while revealing persistent research gaps, including limited geographic coverage for specific taxa (particularly ants in Asia), challenges in rare species handling, and the need for deployment-oriented evaluation frameworks that consider user experience and scalability for citizen science applications. The analysis positions AntID_APP as the first YOLO-based system specifically designed for Taiwan's native ant genera, addressing the geographic and taxonomic gaps identified in the literature.

Table S2. Five-step methodology for acquiring species image datasets through the iNaturalist API.

Steps	URL post example	API usage instructions
(a)	https://api.inaturalist.org/v1/taxa/autocomplete?q=Formicidae	Taxon ID Retrieval: Query the platform using the family name "Formicidae" to obtain its taxonomic ID (1354345).
(b)	https://api.inaturalist.org/v1/observations/species_counts?verifiable=true&taxon_id=47336	Species Enumeration: Using the family ID from (a), retrieve all taxa with available images (4,428 taxa). Each entry contains: Species ID (e.g., 129902) and Scientific name (e.g., Camponotus pennsylvanicus).<br>(Optional: The genus ID (62781) can be obtained by repeating step (a) with "Camponotus")
(c)	https://api.inaturalist.org/v1/observations?verifiable=true&taxon_id=129902&page=1&id_above=286505138	Observation Collection: For each species ID from (b), extract all observations (42,786 total). Each observation provides: Observer ID (e.g., 286616287).<br>(Note: For results exceeding POST limits, use pagination parameters [page, id_below, id_above] for complete retrieval)
(d)	https://api.inaturalist.org/v1/observations/285218198	Image Metadata Extraction: For each observer ID from (c), obtain: Photo metadata: One or more photo IDs (e.g., 512651575) with corresponding URLs and Annotations: Optional observation descriptors including: Alive/Dead status, Evidence of presence, Life stage, Sex.<br>(Annotation availability and categories vary per observation)
(e)	https://inaturalist-open-data.s3.amazonaws.com/photos/512651575/square.jpg	Image Download: Download images using URLs from (d). Available formats: square (75×75 px), small (240 px long edge), medium (500 px long edge), large (1024 px long edge), original (native resolution)<br>(Modify filename in URL to select size)

Details our five-step methodology for systematically acquiring annotated ant image datasets through the iNaturalist API, establishing a reproducible pipeline for ecological computer vision research. The structured workflow comprises: Taxon Identification (Step a), Species Inventory (Step b), Observation Harvesting (Step c), Metadata Enrichment (Step d), and Image Acquisition (Step e). Technical Innovations: (1) Precision Filtering: verifiable=true ensures research-grade quality, (2) Scalability: Handles API pagination limits automatically, and (3) Metadata Preservation: Captures more annotation categories per observation.

Table S3. Best Image Recognition Models Comparison (June 2025):

Category	Model	Strengths	Ideal Use Cases	COCO mAP
**Ultra-High Accuracy**	ConvNeXt-XL<br>Cascade Mask R-CNN	Highest detection accuracy<br>(mAP 62.3)	Medical imaging<br>Satellite remote sensing	62.30%
	SwinV2-G<br>DINOv2	90% accuracy without<br>labeled data	Open-vocabulary detection	60.10%
**Balanced<br>(Speed/Accuracy)**	YOLOv10-X	120 FPS (V100) high mAP	Autonomous driving<br>Surveillance	56.80%
	DETR-ResNet152<br>(2024 Enhanced)	Excels in long-tail data<br>distributions	Retail inventory management	55.20%
**Lightweight Deployment**	MobileOne-Edge<br>(Apple)	>80 FPS on iPhone 14	Mobile apps<br>Drones	48.50%
	NanoDet-Plus	<0.5MB model size	Embedded devices<br>(Raspberry Pi)	45.30%
**Small Object Detection**	YOLOv9-GELAN<br>SPD-Conv	+30% improvement for<br>aerial small objects	Drone inspections	51.70%
**Low-Light Environments**	Zero-DCE++<br>DarkNet-53	+25% mAP in dark scenes	Night surveillance<br>Autonomous vehicles	49.80%
**Multimodal Recognition**	OWL-ViT v2<br>(Google)	Zero-shot detection<br>(text prompts)	Smart assistants<br>Education	58.20%

Evaluates 2025's leading vision models across accuracy (62.3% mAP max), speed (120 FPS), and specialization (30% small-object gains), evaluating performance via COCO mAP and domain-specific capabilities. Highlights include: YOLOv10-X for real-time ecology surveys, YOLOv9-GELAN for insect-sized targets, and OWL-ViT v2 for multimodal queries. The comparative metrics guide model selection for taxonomic applications requiring: (1) micro-scale precision, (2) field deployability, and (3) adaptive learning with limited labeled data.

Table S4. Performance comparison between manual annotation and verification phases

	**Manual Annotation**	**Manual Annotation Verification**	**Increase Multiplier**
**Average Labeling Time (min)**	1,941	142	-13.67
**Average Labeling Accuracy (%)**	86	99	1.15
**Labeling the Number of Pictures**	23,663	52,000	

Performance comparison between manual annotation and verification phases: presents a comparative analysis of efficiency and quality metrics between initial manual annotation and subsequent verification phases in our dataset preparation pipeline. The quantitative comparison reveals three key operational insights: 1. Temporal Efficiency: The verification phase demonstrated dramatic time savings, reducing average processing time from 1,941 minutes (32.35 hours) per batch to just 142 minutes (2.37 hours), representing a 13.67× improvement in throughput. 2. Quality Assurance: Annotation accuracy improved from 86% to 99% post-verification. 3. Scalability Impact: The verified pipeline enabled processing of 52,000 images (2.2× the initial 23,663).

Table S5. YOLO Detection Parameter Taxonomy: Presenting default configurations and their functional definitions across five critical operational categories.

Detection parameter classification	Parameter function definition	Execution parameter preset value
**Screening of trusted objects**	confidence threshold	conf_thres=0.25
	maximum detections per image	max_det=1000
**Detection overlap processing**	NMS IOU threshold	iou_thres=0.45
	class-agnostic NMS	agnostic_nms=False
**Tracking credibility data**	save results to *.txt	save_txt=False
	save confidences in --save-txt labels	save_conf=False
**Improving model reasoning**	augmented inference	augment=False
	visualize features	visualize=False
**Enhancing computing performance**	use FP16 half-precision inference	half=False
	use OpenCV DNN for ONNX inference	dnn=False
	video frame-rate stride	vid_stride=1

The parameter framework is organized as follows: 1. Trusted Object Screening: (1) conf_thres=0.25: Sets the minimum confidence threshold (25%) for initial detection filtering, balancing recall and precision for small entomological targets. (2) max_det=1000: Limits detections per image to prevent memory overload during dense specimen scenarios. 2. Detection Overlap Resolution: (1) iou_thres=0.45: Configures Non-Maximum Suppression (NMS) intersection-over-union threshold to eliminate redundant boxes while preserving closely clustered specimens. (2) agnostic_nms=False: Maintains class-specific suppression to respect taxonomic distinctions during overlap resolution. 3. Data Logging Configuration: save_txt/save_conf=False: Defaults to minimal output formatting, with options to enable confidence score archival for model validation studies. 4. Model Optimization: (1) augment=False: Disables test-time augmentation by default to maintain baseline inference speed. (2) visualize=False: Preserves computational resources by defaulting to minimal feature map visualization. 5. Computational Efficiency: (1) half=False: Conservative FP32 precision default ensures numerical stability for morphological detail preservation. (2) dnn=False: Prioritizes native PyTorch inference over OpenCV DNN backend for compatibility. (3) vid_stride=1: Processes every video frame by default to maximize temporal detection resolution.

Table S6. Quantitative assessment of data augmentation impact on per-genus detection accuracy for 54 ant genera.

No.	Class	[Augmentation] Train/Val/Test: 54000/2700/3729						[No Augmentation] Train/Val/Test: 30573/1547/1348					
		Images	Instances	Box(P)	Box(R)	mAP50	mAP50-95	Images	Instances	Box(P)	Box(R)	mAP50	mAP50-95
-	all	2700	3506	0.930	0.903	0.945	0.804	1547	2072	0.880	0.833	0.907	0.751
1	Acropyga	50	55	0.981	0.942	0.971	0.819	7	10	0.891	0.818	0.959	0.819
2	Aenictus	50	102	0.923	0.818	0.926	0.688	13	37	0.857	0.808	0.887	0.455
3	Anochetus	50	55	0.947	0.982	0.980	0.828	18	23	0.919	0.913	0.981	0.845
4	Anoplolepis	47	79	0.887	0.785	0.870	0.639	16	46	0.835	0.659	0.816	0.531
5	Aphaenogaster	50	50	0.980	0.980	0.989	0.904	50	50	0.923	0.964	0.982	0.909
6	Brachyponera	48	65	0.857	0.800	0.911	0.764	17	34	0.762	0.529	0.677	0.561
7	Camponotus	50	50	0.888	0.820	0.891	0.742	50	50	0.880	0.732	0.872	0.711
8	Cardiocondyla	50	61	0.966	0.933	0.961	0.855	44	55	0.927	0.920	0.972	0.885
9	Carebara	49	81	0.884	0.847	0.904	0.725	25	57	0.840	0.825	0.868	0.695
10	Colobopsis	49	59	0.970	0.966	0.970	0.876	40	50	0.979	0.860	0.960	0.853
11	Crematogaster	50	50	0.991	0.920	0.954	0.862	50	50	1.000	0.837	0.945	0.862
12	Discothyrea	49	119	0.974	0.924	0.952	0.864	4	8	0.779	0.884	0.971	0.795
13	Dolichoderus	50	50	0.933	0.800	0.904	0.783	50	50	0.892	0.800	0.894	0.763
14	Ectomomyrmex	50	50	0.972	0.980	0.982	0.905	10	10	0.863	0.900	0.938	0.855
15	Erromyrma	49	71	0.985	0.952	0.992	0.805	4	4	0.804	0.500	0.825	0.727
16	Formica	50	50	0.805	0.860	0.926	0.591	50	50	0.833	0.797	0.841	0.541
17	Gnamptogenys	50	52	0.987	1.000	0.995	0.855	9	11	0.873	0.909	0.899	0.617
18	Hypoponera	50	51	0.917	0.922	0.971	0.880	25	26	0.838	0.794	0.909	0.830
19	Iridomyrmex	50	102	0.875	0.826	0.867	0.368	50	102	0.896	0.845	0.883	0.388
20	Lasius	50	50	0.915	0.864	0.895	0.715	50	50	0.868	0.800	0.876	0.732
21	Lepisiota	50	100	0.843	0.800	0.897	0.610	40	90	0.817	0.778	0.874	0.537

22	Leptogenys	50	80	0.877	0.805	0.891	0.745	50	80	0.962	0.738	0.909	0.756
23	Lioponera	50	51	1.000	0.944	0.994	0.855	8	9	1.000	0.978	0.995	0.779
24	Lophomyrmex	48	70	0.971	0.940	0.973	0.825	5	6	0.819	1.000	0.995	0.807
25	Meranoplus	50	63	1.000	0.924	0.986	0.865	50	63	1.000	0.921	0.988	0.875
26	Messor	50	50	0.928	0.900	0.943	0.810	50	50	0.927	0.920	0.939	0.800
27	Monomorium	50	50	0.863	0.880	0.934	0.879	50	50	0.859	0.880	0.922	0.845
28	Myrmecina	48	48	0.993	0.958	0.961	0.861	10	10	0.894	0.700	0.878	0.771
29	Myrmica	50	50	0.959	0.980	0.989	0.932	50	50	0.930	0.940	0.975	0.920
30	Nylanderia	50	58	0.865	0.759	0.858	0.714	50	58	0.813	0.749	0.843	0.733
31	Ochetellus	50	112	0.962	0.955	0.974	0.815	6	10	0.922	0.600	0.698	0.413
32	Odontomachus	50	50	1.000	0.995	0.995	0.888	50	50	0.961	1.000	0.995	0.892
33	Odontoponera	48	49	0.961	0.980	0.975	0.867	7	8	0.964	1.000	0.995	0.888
34	Ooceraea	49	83	0.946	0.964	0.982	0.754	6	10	0.767	0.800	0.856	0.582
35	Paraparatrechina	47	52	0.996	0.923	0.950	0.836	10	15	1.000	0.605	0.833	0.678
36	Paratopula	49	50	0.979	0.948	0.976	0.909	6	6	0.961	1.000	0.995	0.977
37	Parvaponera	47	47	0.985	0.957	0.977	0.906	4	4	0.910	1.000	0.995	0.948
38	Pheidole	50	50	0.885	0.880	0.929	0.843	50	50	0.840	0.820	0.906	0.805
39	Plagiolepis	49	77	0.750	0.779	0.825	0.551	34	62	0.767	0.806	0.818	0.518
40	Polyrhachis	50	50	0.925	0.940	0.979	0.890	50	50	0.934	0.980	0.979	0.914
41	Ponera	49	55	0.956	0.945	0.971	0.889	18	24	0.843	0.893	0.878	0.786
42	Pristomyrmex	49	57	0.930	0.912	0.955	0.847	10	17	0.833	0.706	0.849	0.764
43	Proceratium	50	50	1.000	0.934	0.972	0.850	7	7	0.911	1.000	0.995	0.827
44	Pseudoponera	49	50	0.994	0.980	0.989	0.886	4	4	0.667	1.000	0.945	0.786
45	Solenopsis	50	50	0.976	0.802	0.926	0.811	50	50	0.876	0.800	0.889	0.790
46	Stigmatomma	50	50	0.986	1.000	0.995	0.890	9	9	0.802	0.778	0.918	0.833

47	Strumigenys	50	50	0.952	0.940	0.983	0.879	50	50	0.959	0.944	0.978	0.859
48	Tapinoma	50	83	0.647	0.818	0.772	0.607	35	68	0.607	0.727	0.742	0.603
49	Technomyrmex	50	154	0.849	0.764	0.890	0.768	43	147	0.899	0.707	0.814	0.710
50	Temnothorax	50	50	0.923	0.956	0.967	0.926	50	50	0.875	0.979	0.933	0.874
51	Tetramorium	50	50	0.839	0.833	0.925	0.752	50	50	0.938	0.880	0.927	0.743
52	Tetraponera	49	51	0.938	0.884	0.958	0.821	34	36	0.939	0.860	0.935	0.820
53	Trichomyrmex	49	53	0.909	0.941	0.959	0.788	12	16	0.878	0.750	0.914	0.586
54	Vollenhovia	49	161	0.987	0.935	0.965	0.871	7	40	0.962	0.637	0.898	0.750

This table presents a comparative analysis of per-genus detection performance under two training configurations: (1) with the full augmentation pipeline (54,000 training images), and (2) without augmentation (30,573 training images). For each genus, the following metrics are reported: number of original training images (Images), number of instances after augmentation (Instances), bounding box precision (Box(P)), bounding box recall (Box(R)), mean Average Precision at IoU threshold 0.5 (mAP50), and mean Average Precision across IoU thresholds from 0.5 to 0.95 (mAP50-95). The results reveal considerable variation in generalization capability across genera. While the overall mAP50 averages 0.945, per-genus mAP50-95 values range from 0.368 (Iridomyrmex) to 0.932 (Myrmica), indicating that some genera with limited original samples may be affected by overfitting to augmentation artifacts. This granular analysis provides transparency regarding model robustness and informs future efforts to improve classification reliability for underrepresented taxa.

Table S7. Key Hyperparameters for YOLO Model Training and Tuning

Category	YOLO-style Parameter (Common Value Range / Options)
**Dataset & Core Settings**	data (path/to/data.yaml); epochs (100 - 300);<br>batch-size (16, 32, 64); imgsz / img (320, 640, 1280)
**Model Architecture**	cfg (yolo11s.yaml, yolo10m.yaml)
**Optimizer & Learning Rate (Most Critical)**	optimizer (SGD, Adam, AdamW);<br>lr0 (SGD: 0.01, Adam: 0.001); lrf (0.01, 0.1);<br>momentum (0.9 - 0.98); weight_decay (5e-4)
**Data Augmentation (Improves Generalization)**	hsv_h, hsv_s, hsv_v (0.015, 0.7, 0.4); translate (0.0 - 0.2);<br>scale (0.5 - 0.9); flipud, fliplr (0.0 - 0.5); mosaic (0.0 - 1.0)
**Other Important**	patience (50 - 100); device (0, 1); workers (4 - 8)

This table provides a comprehensive comparison of four medium-scale YOLO model variants (yolov9m, yolo10m, yolo11m, and yolo12m), evaluating their performance across key metrics of accuracy, model complexity, and computational efficiency. 1. Dataset & Core Settings: data: Path to the dataset config file (data.yaml), defining paths and class names. epochs: Number of complete training cycles through the entire dataset. batch-size: Number of images processed per gradient update (limited by GPU memory). imgsz: Input image size (e.g., 640). Larger sizes can improve accuracy but are slower. 2. Model Architecture: cfg: The model architecture definition (e.g., yolo11s.yaml for a "small" model). 3. Optimizer & Learning Rate: optimizer: Algorithm for weight updates (e.g., SGD or Adam). lr0: Initial Learning Rate. The single most important parameter to tune. lrf: Ratio of the final learning rate to the initial rate. momentum: Helps accelerate SGD and overcome local minima. weight_decay: Regularization technique to prevent overfitting. 4. Data Augmentation: hsv_h/s/v: Adjusts image hue, saturation, and value to simulate lighting changes. translate: Fraction of image width/height to randomly translate. scale: Fraction to randomly scale the image. flipud, fliplr: Probability of vertical/horizontal flip. mosaic: Probability of applying mosaic augmentation (combines 4 images). 5. Other Important: patience: Number of epochs to wait for improvement before stopping early. device: Computing device to use for training. workers: Number of subprocesses for data loading.

Table S8. Performance and Hyperparameter Comparison of YOLO Architectures (v9-v12) (Nano-XLarge) Under GPU Memory Constraints.

model	mAP50	mAP50-95	precision	recall	time(hrs)	batch	optimizer	lr0	lrf	GFLOPs
YOLOv9m	0.931	0.783	0.916	0.884	67.573	32	SGD	0.01	0.01	77.8
YOLOv9c	0.937	0.790	0.934	0.880	83.079	16	SGD	0.01	0.01	103.9
YOLO10l	0.948	0.807	0.929	0.902	86.358	16	SGD	0.01	0.01	127.7
YOLO11n	0.886	0.691	0.885	0.808	26.555	16	SGD	0.01	0.01	6.5
YOLO11l	0.947	0.802	0.932	0.900	75.073	16	SGD	0.01	0.01	87.5
YOLO11m	0.935	0.779	0.927	0.897	55.354	32	SGD	0.01	0.01	68.4
YOLO11m	0.936	0.773	0.935	0.873	63.509	8	SGD	0.01	0.01	68.4
YOLO11m	0.882	0.692	0.874	0.802	56.631	16	Adam	0.001	0.01	68.4
YOLO11m	0.901	0.740	0.890	0.832	58.509	16	AdamW	0.001	0.01	68.4
YOLO11m	0.893	0.704	0.878	0.817	54.991	32	Adam	0.001	0.01	68.4
YOLO11m	0.912	0.742	0.920	0.837	55.378	32	AdamW	0.001	0.01	68.4

This table compares the performance of YOLO architectures from v9 to v12 (Nano to XLarge) under maximum GPU memory constraints, evaluating mAP50, mAP50-95, precision, recall, training time, and GFLOPs. It focuses on the impact of key hyperparameters: batch size, optimizer, and learning rate. 1. Model Performance & Scalability: A clear trade-off exists between model size and performance. YOLO10l achieves the highest accuracy (mAP50: 0.948, mAP50-95: 0.807), while the compact YOLO11n has the lowest computational cost (6.5 GFLOPs) and shortest training time (26.6h), albeit with the lowest accuracy (mAP50: 0.886). Models like YOLOv9c and YOLO11l offer a balanced midpoint. 2. GPU Memory Limitations: Several large models (YOLOv9e, YOLO10x, YOLO11x, YOLO12l) exceeded GPU memory limits and could not be trained, though they are theorized to offer higher performance. 3. Hyperparameter Insights: (1) Batch Size: Increasing the batch size from 16 to 32 for models like YOLOv9m and YOLO11m slightly improved mAP50-95 and recall while reducing training time, demonstrating benefits from more stable gradients. (2) Optimizer: SGD outperformed adaptive optimizers (Adam/AdamW) for YOLO11m, aligning with its known generalization strength for object detection. At a batch size of 16, SGD achieved an mAP50 of 0.936, compared to 0.882 for Adam and 0.901 for AdamW. (3) Learning Rate: Using a high initial learning rate (0.01) with Adam/AdamW caused training failure, confirming that these optimizers require lower rates (e.g., 0.001) for

stability. (4) Memory Failures: YOLO10m and YOLO12m encountered out-of-memory errors at a batch size of 32, underscoring the hardware constraints that shape hyperparameter choices.

Table S9. Cross-domain generalization performance on an external test set of 1,458 ant images from diverse sources.

NO.	Genus Name	Images	Correct	Instances	Accuracy
1	Acropyga	12	8	10	0.667
2	Aenictus	7	4	12	0.571
3	Anochetus	15	12	13	0.800
4	Anoplolepis	21	11	12	0.524
5	Aphaenogaster	67	51	63	0.761
6	Brachyponera	16	15	19	0.938
7	Camponotus	100	83	93	0.830
8	Cardiocondyla	4	4	4	1.000
9	Carebara	14	11	62	0.786
10	Colobopsis	17	16	20	0.941
11	Crematogaster	102	86	132	0.843
12	Discothyrea	13	12	16	0.923
13	Dolichoderus	51	45	50	0.882
14	Ectomomyrmex	0	0	0	-
15	Erromyrma	0	0	0	-
16	Formica	108	91	105	0.843
17	Gnamptogenys	6	4	4	0.667
18	Hypoponera	20	17	21	0.850
19	Iridomyrmex	42	35	68	0.833
20	Lasius	64	44	53	0.688
21	Lepisiota	7	5	6	0.714
22	Leptogenys	25	24	34	0.960
23	Lioponera	7	7	14	1.000
24	Lophomyrmex	0	0	0	-
25	Meranoplus	10	9	15	0.900
26	Messor	1	1	1	1.000
27	Monomorium	43	30	40	0.698
28	Myrmecina	3	3	4	1.000
29	Myrmica	21	20	21	0.952
30	Nylanderia	45	44	73	0.978
31	Ochetellus	14	7	15	0.500
32	Odontomachus	70	64	71	0.914
33	Odontoponera	0	0	0	-
34	Ooceraea	3	2	3	0.667
35	Paraparatrechina	4	3	3	0.750
36	Paratopula	0	0	0	-
37	Parvaponera	0	0	0	-

38	Pheidole	100	72	83	0.720
39	Plagiolepis	0	0	0	-
40	Polyrhachis	29	24	30	0.828
41	Ponera	5	5	5	1.000
42	Pristomyrmex	3	2	2	0.667
43	Proceratium	14	12	12	0.857
44	Pseudoponera	4	3	3	0.750
45	Solenopsis	100	73	90	0.730
46	Stigmatomma	24	17	28	0.708
47	Strumigenys	57	55	69	0.965
48	Tapinoma	35	28	56	0.800
49	Technomyrmex	20	18	123	0.900
50	Temnothorax	54	39	45	0.722
51	Tetramorium	57	44	49	0.772
52	Tetraponera	16	16	19	1.000
53	Trichomyrmex	2	1	1	0.500
54	Vollenhovia	6	5	13	0.833

To assess whether the model generalizes beyond iNaturalist-style imagery, we constructed an external test set comprising 1,458 ant images sourced from Google Images (n=875), Flickr (n=365), and manually photographed field specimens under uncontrolled conditions (n=218). This external set was designed to represent greater diversity in photographic conditions, including variable lighting, backgrounds, camera hardware, and image compression artifacts, while excluding any images overlapping with the original iNaturalist training data. For each genus with available test samples (Images > 0), the table reports: the number of test images (Images), the number of correctly identified images at the genus level (Correct), the number of ground truth instances (Instances), and the genus-level identification accuracy (Accuracy = Correct / Images). Genera with zero test images (e.g., Ectomomyrmex, Erromyrma, Lophomyrmex, Odontoponera, Paratopula, Parvaponera, Plagiolepis) are excluded from this analysis. The overall mAP50 achieved by the YOLO11m model on this external set was 0.811, representing a 13.3% performance decline compared to the 0.936 mAP50 obtained on the iNaturalist test set. This performance gap quantifies the domain shift between curated citizen science imagery and uncontrolled internet-sourced photographs. Among genera with sufficient test samples ($n \geq 10$), those with distinctive morphological features (e.g., Odontomachus: 0.914, Strumigenys: 0.965) maintained relatively high accuracy, while morphologically conservative genera (e.g., Tapinoma: 0.800, Tetramorium: 0.772) exhibited lower accuracy, indicating greater sensitivity to domain shift. Notably, some genera with limited test samples (e.g., Cardiocondyla: 1.000, Lioponera: 1.000, Messor: 1.000, Ponera: 1.000, Tetraponera: 1.000) achieved perfect accuracy, though these results should be interpreted with caution due to small sample sizes.

```
Python 3.9.12 (main, Apr  4 2022, 05:22:27) [MSC v.1916 64 bit (AMD64)]
Type "copyright", "credits" or "license" for more information.

IPython 8.2.0 -- An enhanced Interactive Python.

In [1]: runfile('D:/202506/SpeciesID.py', args='species Formicidae', wdir='D:/202506')
[                                                  ] 100% 4405/4405 0:49:48
Collect Total Species:4405  Exclude Not Species:19
```

Figure S1. Runtime output screenshot of the Collect program showing: (a) input parameters (function=species, family_name=Formicidae), (b) execution path (D:/202506), and (c) results summary (4,405 species collected with 19 non-species records). Shows the console output of our taxonomic collection script, confirming successful retrieval of 4,405 ant species records (Formicidae) with 19 invalid entries filtered (99.57% accuracy). The 49-minute runtime demonstrates efficient batch processing capability. Annotated components include input parameters, execution path, and validation metrics, establishing a reproducible workflow for large-scale biodiversity data acquisition.

	A	B	C	D	E	F	G
1	spatial:						
2	Affine	1					p
3	Crop	1	0	0	400	400	p, x_min, y_min, x_max, y_max
4	ElasticTransform	1	1	50	50		p, alpha, sigma, alpha_affine
5	pixel:						
6	Blur	1	7				p, blur_limit
7	ChannelDropout	1	0	(1, 1)			p, fill_value, channel_drop_range
8	Defocus	1	(1, 7)	(0.1, 0.5)			p, radius, alias_blur

Figure S2. Albumentations configuration file: Presents the configuration framework for the Albumentations data augmentation pipeline used in our image recognition system. The table details two primary categories of transformations with their respective parameter settings: Spatial-level Transformations: (1) Affine: Applied with probability p=1 (always active), enabling rotation, scaling, and shearing while preserving spatial relationships. (2) Crop: Configured with 400×400 pixel dimensions (x_min/y_min to x_max/y_max) and probability p=1. (3) ElasticTransform: Set with α=50 (deformation intensity), σ=50 (smoothness), and α_affine=50 (affine transformation intensity).
Pixel-level Transformations: (1) Blur: Implemented with 7×7 kernel size (blur_limit) and p=1. (2) ChannelDropout: Configured to drop single channels (channel_drop_range=(1,1)) while maintaining fill_value=0. (3) Defocus: Parameterized with radius range (1-7 pixels) and alias_blur range (0.1-0.5) to simulate optical aberrations

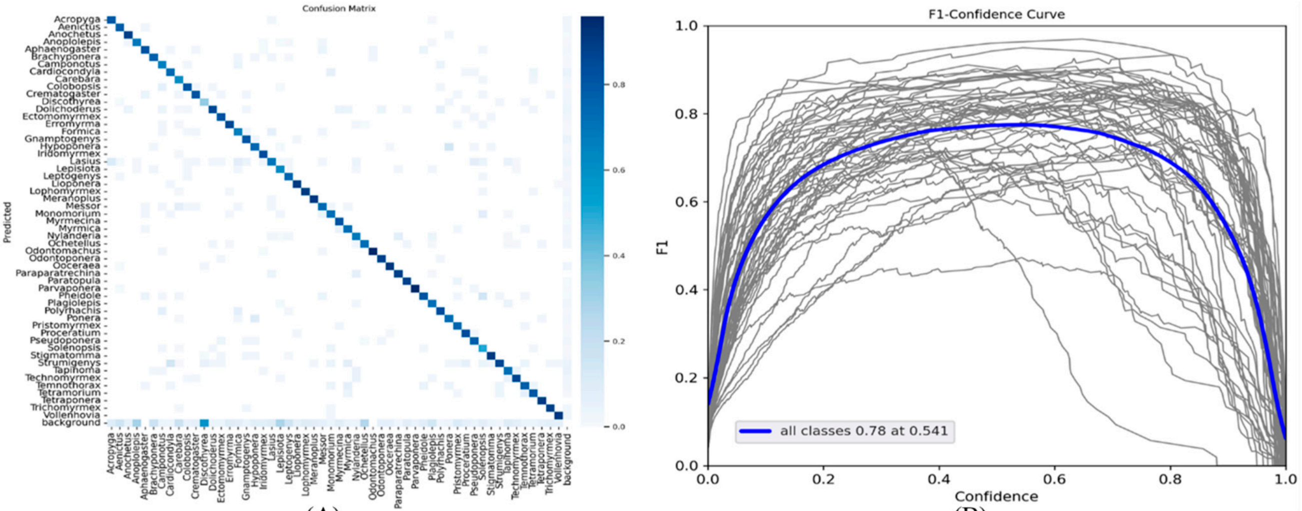

(A) (B)

Figure S3. Comparative Analysis of Taxonomic Classification Performance: Consists of two key analytical components: (A) Genus-Level Confidence Distribution (Confusion Matrix): This panel visualizes the distribution of confidence scores across different ant genera, highlighting the model's reliability at the genus level. The box plots (or density distributions) reveal variations in prediction certainty, with annotated outliers indicating challenging taxa or ambiguous morphological features. This analysis helps identify systematic biases or taxonomic groups requiring model refinement. (B) F1-Score Confidence Threshold Optimization: This panel demonstrates the trade-off between precision and recall across different confidence thresholds. The plotted F1-score curve identifies the optimal threshold (marked with a dashed line) that maximizes harmonic mean performance. Annotations compare baseline metrics (e.g., default threshold at 0.5) with the optimized value, emphasizing its impact on reducing false positives while retaining true identifications. Together, these analyses provide empirical insights into model calibration and decision-boundary tuning, critical for deploying robust species identification systems in ecological research.

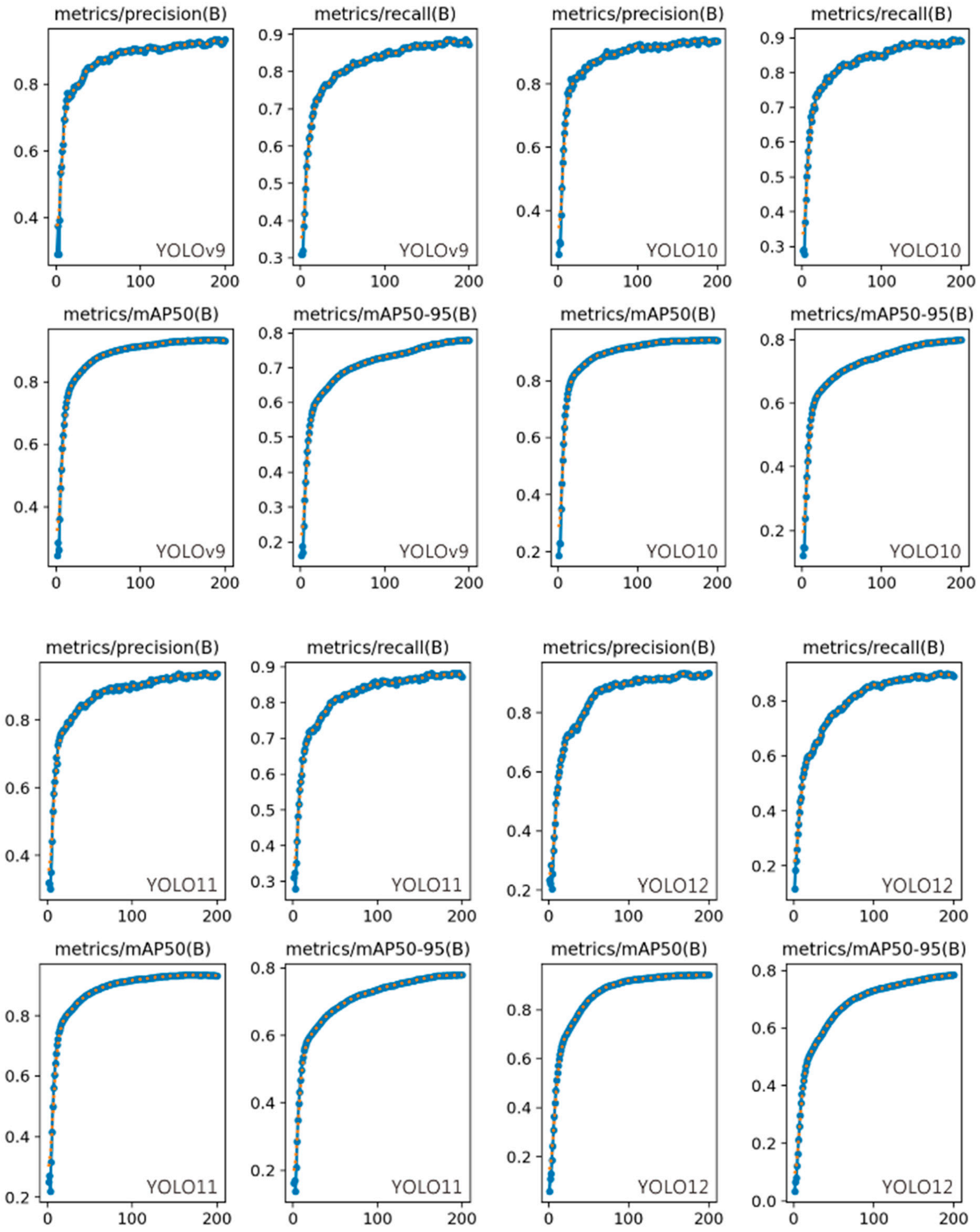

Figure S4. Training Curves of Key Metrics: mAP50, mAP50-95, Precision, and Recall for YOLO Models (YOLOv9m, YOLOv10m, YOLOv11m, and YOLOv12m) Across 200 Epochs.

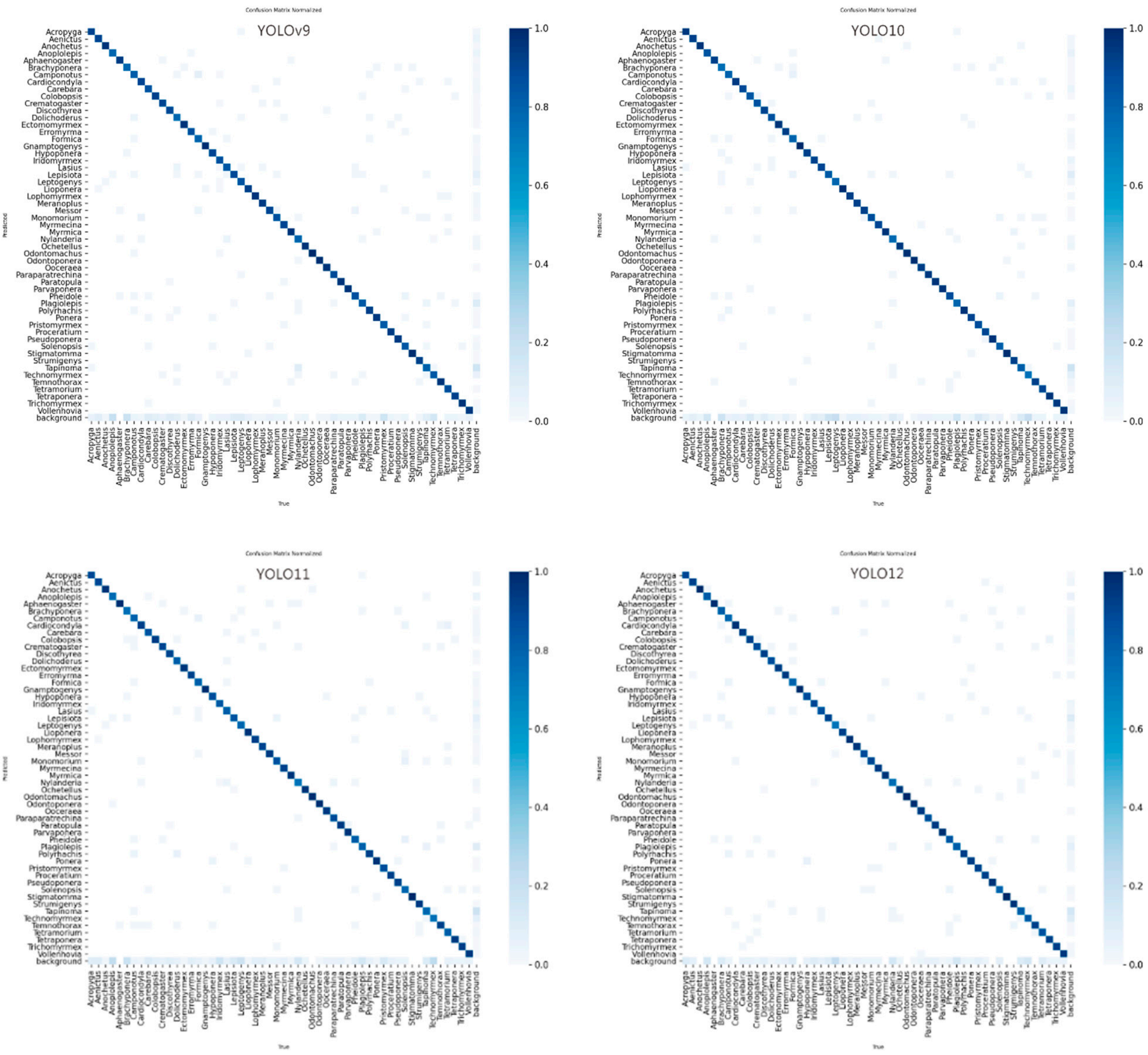

Figure S5. Normalized Confusion Matrices for YOLO Model Variants (v9m, v10m, v11m, v12m): 1. Evolution of Precision: v12m demonstrates the cleanest diagonal overall, with fewer intense off-diagonal errors, suggesting better feature disentanglement. v9m shows slightly higher confusion in fine-grained classes. 2. Localization vs. Classification Trade-offs: v9m and v10m prioritize localization accuracy, occasionally at the cost of minor classification errors. v11m and v12m show more balanced localization-classification performance, resulting in tighter diagonal concentrations. 3. Error Distribution: v9m and v10m exhibit broader error spread across multiple classes. v11m and v12m errors are more concentrated toward specific challenging pairs, indicating targeted improvements.

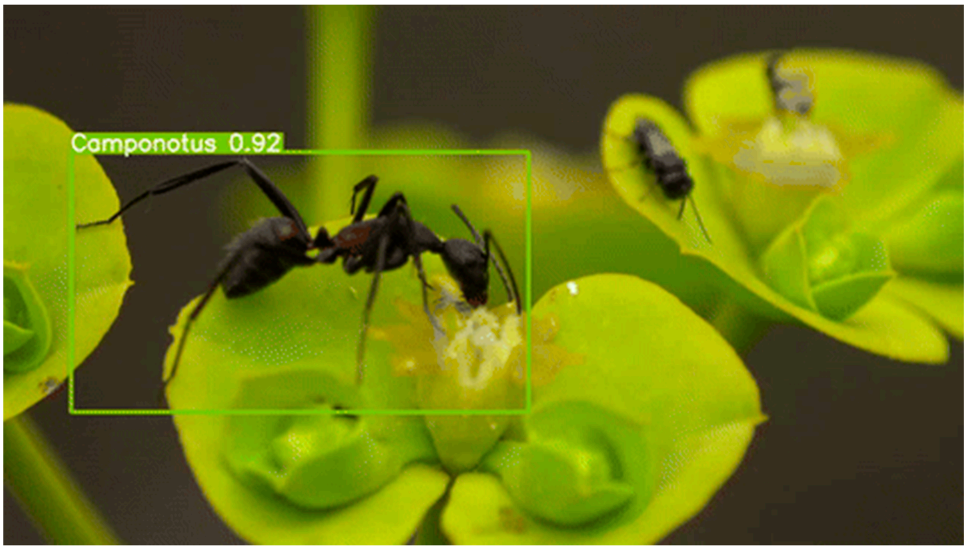

Figure S6. Animated demonstration of video frame-based ant identification using AntID_APP: This animated GIF illustrates the process of extracting and identifying ant specimens from video footage. The sequence shows 24 consecutive frames per second from a video recording of Camponotus sp., with each frame processed in real-time by the YOLO-based detection model. The green bounding boxes indicate successful ant detection, and the confidence scores (>0.95 across all frames) demonstrate the model's robustness to minor variations in ant posture, lighting, and background within the video stream. This capability enables citizen scientists to utilize video recordings—rather than static images—for species identification, significantly increasing the volume of usable field data. The corresponding static frame used in Figure 6C is extracted from this video sequence. For optimal viewing, please use a GIF-compatible viewer.
